# 3D Porous Collagen Matrices—A Reservoir for In Vitro Simultaneous Release of Tannic Acid and Chlorhexidine

**DOI:** 10.3390/pharmaceutics15010076

**Published:** 2022-12-26

**Authors:** Lavinia Brăzdaru, Teodora Staicu, Mădălina Georgiana Albu Kaya, Ciprian Chelaru, Corneliu Ghica, Viorel Cîrcu, Minodora Leca, Mihaela Violeta Ghica, Marin Micutz

**Affiliations:** 1 Department of Physical Chemistry, University of Bucharest, 4-12 Regina Elisabeta Blvd., 030018 Bucharest, Romania; 2 Leather and Footwear Research Institute, 93 Ion Mincu St., 031215 Bucharest, Romania; 3 National Institute of Materials Physics, 105 bis Atomistilor St., 077125 Magurele, Romania; 4 Department of Inorganic Chemistry, University of Bucharest, 4-12 Regina Elisabeta Blvd., 030018 Bucharest, Romania; 5 Faculty of Pharmacy, University of Medicine and Pharmacy “Carol Davila”, 6 Traian Vuia St., 020956 Bucharest, Romania; 6 Institute of Physical Chemistry “Ilie Murgulescu”, Romanian Academy, 202 Spl. Independenţei, 060021 Bucharest, Romania

**Keywords:** collagen hydrogels, porous collagen matrices, tannic acid release, chlorhexidine release

## Abstract

The treatment of wounds occurring accidentally or as a result of chronic diseases most frequently requires the use of appropriate dressings, mainly to ensure tissue regeneration/healing, at the same time as treating or preventing potential bacterial infections or superinfections. Collagen type I-based scaffolds in tandem with adequate antimicrobials can successfully fulfill these requirements. In this work, starting from the corresponding hydrogels, we prepared a series of freeze-dried atelocollagen type I-based matrices loaded with tannic acid (*TA*) and chlorhexidine digluconate (*CHDG*) as active agents with a broad spectrum of antimicrobial activity and also as crosslinkers for the collagen network. The primary aim of this study was to design an original and reliable algorithm to in vitro monitor and kinetically analyze the simultaneous release of *TA* and *CHDG* from the porous matrices into an aqueous solution of phosphate-buffered saline (*PBS*, pH 7.4, 37 °C) containing micellar carriers of a cationic surfactant (hexadecyltrimethylammonium bromide, *HTAB*) as a release environment that roughly mimics *human extracellular fluids* in living tissues. Around this central idea, a comprehensive investigation of the lyophilized matrices (morpho-structural characterization through FT-IR spectroscopy, scanning electron microscopy, swelling behavior, resistance against the collagenolytic action of collagenase type I) was carried out. The kinetic treatment of the release data displayed a preponderance of non-Fickian–Case II diffusion behavior, which led to a general anomalous transport mechanism for both *TA* and *CHDG*, irrespective of their concentrations. This is equivalent to saying that the release regime is not governed only by the gradient concentration of the releasing components inside and outside the matrix (like in ideal Fickian diffusion), but also, to a large extent, by the relaxation phenomena of the collagen network (determined, in turn, by its crosslinking degree induced by *TA* and *CHDG*) and the dynamic capacity of the *HTAB* micelles to solubilize the two antimicrobials. By controlling the degree of physical crosslinking of collagen with a proper content of *TA* and *CHDG* loaded in the matrix, a tunable, sustainable release profile can be obtained.

## 1. Introduction

An almost ideal wound dressing must meet some mandatory features: ease of application, bioadhesiveness to the wound surface, absorption of excess exudates, maintenance of a moist wound environment, provision of thermal insulation and bacterial barrier, compatibility with topical therapeutic agents, capacity to release them, ease of sterilization, non-toxic and non-antigenic properties, and biodegradability [1,2].

Collagen matrices fulfill all the above requirements [2,3,4,5,6]. Moreover, they are active elements of wound management. Given their three-dimensional structure, with large and interconnected pores, collagen matrices build up an optimum microenvironment for healing the wound, stimulating the development of new tissue, cell adhesion, and formation of new granulation tissue and epithelium on the wound [5,7]. Even though collagen, as a porous matrix, exhibits an important role in promoting the healing process of an injured tissue (together with other components, such as growth factors), unfortunately, it serves as substrate for the development of bacteria as well. However, such collagen-based matrices can be used as a physical reservoir and substrate in controlling wound infection by the localized delivery of antimicrobials [7,8,9,10].

One of the oldest antimicrobials used for wound healing, especially burns, is tannic acid (*TA*) [11,12,13]. Due to the large number of free phenolic hydroxyl groups, *TA* forms strong hydrogen bonds with proteins, resulting in their physical crosslinking [14,15,16]. On the other hand, *TA*, as a specific representative of natural tannins, may interact with collagen not only by hydrogen bonding [17,18], but also hydrophobically [18,19,20], with the formation of tannin–protein complexes [20]. By choosing *TA* as a crosslinking agent for collagen hydrogels or collagen-based dry matrices, the potential cytotoxicity produced by the classical chemical crosslinkers [21] can thus be avoided, even though there is also the approach of obtaining real hydrogels covalently crosslinked under the action of ionizing radiation (γ rays, accelerated electron beams) in the absence of any crosslinking molecules when it comes to the synthesis of collagen-based hydrogels [22,23,24], or, generally, of other types of polymer/biopolymer hydrogels [25,26]. Starting from the general crosslinking ability reported in building up 3D polymeric networks [27], *TA* has proved to be a very promising partner in *TA*-crosslinked collagen scaffolds intended for many biomedical purposes [27,28,29,30,31,32,33], and, very recently, even in attempting to synthesize a solid catalyst consisting of collagen fibers loaded with *TA*, Fe^3+^, and peroxymonosulfate, with excellent properties of adsorption towards rhodamine B (via π–π interactions between *TA* and the organic dye) and chemical affinity to decompose it (by facilitating redox cycle Fe^3+^-Fe^2+^ under the reducing activity of *TA* to finally promote production of sulfate radicals from peroxymonosulfate effective in dye decay), and, eventually, dedicated to treating the industrial wastewater polluted with aromatic-type organic dyes [33].

In fact, the role played by *TA* in such *TA*–collagen mixed systems is of a relatively efficient crosslinker for collagen molecules (demonstrated by the increase of several degrees in the denaturation temperature of collagen belonging to the *TA*-crosslinked matrices compared to that of collagen in uncrosslinked samples, together with a similar tendency in the resistance against the collagenolytic effect of the specific enzymes [27,32,34]) on one hand, and a potential therapeutic agent progressively released into the surrounding aqueous environment [28,29,30,32,35] on the other hand. Thus, a number of interesting studies conducted on collagen–*TA* matrices during the last few years have revealed useful applications in the biomedical field, mainly involving biocompatible hydrogels with antimicrobial characteristics [29] or *TA*-crosslinked collagen scaffolds related to malignant diseases (particularly breast cancer): for example, cell scaffolds as a subdermal tissue regenerative matrix for breast reconstruction [28,30], or, in line with this aspect, biocompatible scaffolds releasing *TA* proved to induce apoptosis in ER^+^ breast cancer cells (MCF-7 cells) [32,35]. At the same time, *TA* alone was found to be an effective antifibrotic compound in pulmonary fibrosis, mainly by inhibiting the activity of the growth factor TGFβ responsible, among other things, for the overexpression of collagen type I deposited into the extracellular matrix of the lung tissue [31].

Medical use of such antimicrobials extracted from plants, such as tannic acid, is frequently associated with synthetic antimicrobials. Among the latter, chlorhexidine digluconate (*CHDG*) is the most utilized biocide in clinical practice for skin antisepsis and in oral care products, being considered the gold standard for plaque control and for treating or preventing periodontal diseases in order to maintain oral cavity health [36,37,38,39,40,41,42]. In fact, the broad-spectrum antimicrobial bioactivity of chlorhexidine (*CH*) has been reported in countless studies over a long period of time, more than six decades, but recent works are focused on designing novel *CH*-loaded carriers/materials/composites able to supply topically a tunable amount of active component (*CH*–copper composite as micrometer sized particles with synergetically enhanced activity compared to that silver particles [43]; *CH*-loaded biocompatible gelatin fibers with antimicrobial activity demonstrated against both Gram-negative and Gram-positive bacteria [44]; *CH*-loaded nanocarriers—*CH*-containing shellac nanoparticles exhibiting enhanced bioactivity of *CH* [45], *CH*-loaded copolymer nanogel carriers superficially functionalized with a cationic polyelectrolyte with amplified and broad biocidal action [46], nanoparticles of amorphous calcium phosphate containing *CH* with a beneficial action of inhibiting collagen type I degradation and promoting its mineralization at the adhesive–dentin interface, with a direct consequence in slowing down the aging of the resin–dentine contact [47]; bioresorbable polylactide-based and collagen-based membranes previously soaked in *CHDG* solution displaying a certain retardation of bacterial colonization when the membranes were exposed to oral activity [48]). In addition, other relatively newly obtained results deal specifically with *CH* action in treatment concerning curli production and biofilm formation (*E. colli*) [49], with new formulations of *CH*-based mouth rinse with bioactivity against pathogenic bacteria such as *P. mutans* and *P. gingivalis* [50], or in the wound healing of human gingival tissue that has been post-surgically affected [36]. Last but not least, other works proved that some enzymatically treated undesirable oral biofilms can be disrupted and removed under the action of low concentrations of aqueous *CHDG* solutions (<0.1%) [37], and, what is truly noteworthy, the bacteria (*P. aeruginosa*, *S. aureus*) killed by *CHDG* may behave similarly to zombie cells, which possess high biocidal activity against the next generation of viable bacteria [51].

In some circumstances, the biocidal effect of *CH* may be potentiated significantly by using it together with some phytochemicals [38,39,52]. Similar synergistic effects were also reported for other types of synthetic antimicrobials [53]. There are practical results that show a direct influence of some phytochemicals on transforming the antimicrobial characteristics of *CHDG* from bacteriostatic to bactericidal [39]. To the best of our knowledge, there are very few data on mixed *CH–TA* systems [54], even if oral care products containing both *CH* and *TA* are already in use [55].

Based on the antimicrobial potential of *TA* and *CHDG*, we report in the present paper some results focused on several peculiar aspects, mainly regarding both the characterization of multicomponent *TA–CHDG*–collagen systems and the simultaneous release of *TA*/*CHDG* in an aqueous physiological-like medium. Firstly, the effect of different compositions of *TA*–*CHDG* on the native conformation of collagen in the initial *TA*–*CHDG*-collagen matrices was assessed by FT-IR spectroscopy. Then, the morphological changes induced by the presence of *TA*–*CHDG* in these freeze-dried *TA*–*CHDG*–collagen sponges were revealed by employing SEM. At the same time, the prepared porous collagen-based matrices in the dry state were characterized by swelling capacity in a phosphate-buffered saline (*PBS*) solution and also by their resistance to collagenase digestion in a phosphate buffer of pH 7.4. Eventually, we spectrophotometrically (UV–VIS) monitored and kinetically studied the simultaneous release of *TA* and *CHDG* from the collagen-based matrices in a *PBS* solution (pH 7.4) containing cationic micellar carriers.

## 2. Materials and Methods

### 2.1. Materials

The collagen type I (extracted from bovine skin) hydrogel of 2.50% (by weight), pH 3.20, was supplied by the National Research-Development Institute for Textiles and Leather, Division Leather and Footwear Research Institute–NRDITL-LFRI, Romania. *TA* (powder, ACS reagent), collagenase type I (lyophilized powder, from *Clostridium histolyticum*), paraffin wax, and petroleum ether (ACS reagent) were acquired from Sigma-Aldrich. Pyrene and hexadecyltrimethylammonium bromide (*HTAB*) from Fluka, *CHDG* (20% aqueous solution, by weight) from Caesar & Loretz GmbH (Hilden, Germany), and ethanol (ACS reagent absolute, ≥99.5%) from EMPARTA were used as received. All the other reagents and chemicals were purchased from Reactivul–Bucharest, Romania and utilized without further processing.

### 2.2. Preparation of Collagen Hydrogels and Matrices

Acidic collagen hydrogels (pH of about 4) with and without *TA* and *CHDG* were prepared as described elsewhere [56]. Briefly, a series of nine collagen hydrogels (pH 4) of 1.10% collagen and different contents of *TA* and *CHDG* (by taking into account all combinations of 5, 10, 15% *TA* and 1.82, 4.55, 9.09% *CHDG*, the concentrations being considered with respect to the collagen) were prepared by adding proper amounts of aqueous solutions of *TA* and/or *CHDG* to a certain quantity of initial collagen hydrogel (2.50% and pH 3.20) under gentle stirring. To maintain the overall pH of the systems at ca. 4 during the collagen-based hydrogels preparation, a solution of 1M NaOH was used. A series of three collagen hydrogels containing *TA* (5, 10, and 15%) for comparison were also prepared. All the collagen-based hydrogels were equilibrated by keeping them at 4 °C for 24 h. The final porous collagen-based matrices were obtained by freeze-drying collagen-based hydrogels (poured into molds with a flat bottom and open to the air side on top) for 48 h, employing a Delta 2–24 LSC (Martin Christ, Osterode am Harz, Germany) freeze-dryer. A detailed algorithm of the entire lyophilization process was described previously [57].

### 2.3. Matrices Characterization

#### 2.3.1. FT-IR Measurements

Structural characterization of collagen from collagen-based matrices was performed by FT-IR using a JASCO FT-IR 4200 spectrometer equipped with a TGS detector and Spectra Manager software. Data were acquired by ATR technique using a MKII Golden Gate 45 degree diamond crystal with a horizontal ATR plate. All the spectra were corrected for ATR effect and transformed into absorption spectra. Each spectrum is the average of 30 scans, with a resolution of 4 cm^−1^.

#### 2.3.2. Morphology Investigations

Morphologies of dry porous collagen-based matrices were examined by scanning electron microscopy. A metal coating applied to a freshly sectioned area of a matrix makes the sample conductive and ready to expose to the electron beam of the microscope gun with no significant damages. The metal deposition (Au–99.99%, normal incidence) step took place by metal vaporization under high vacuum (by resistive heating of a tungsten filament under a high vacuum of 5 × 10^−6^ torr) inside an evaporator Jeol-JEE 4C, as described in another paper [58]. The SEM images were acquired by employing a microscope Tescan Lyra 3 XMU FEG at an acceleration voltage of 5 kV.

#### 2.3.3. UV–VIS Absorption Spectroscopy and Steady-State Fluorescence Measurements

In order to quantify the amounts of *TA* and *CHDG* released as a function of time, UV–VIS absorption spectra were recorded by using a Jasco—V660 spectrophotometer equipped with a thermostating module.

Steady-state fluorescence of pyrene was considered to be useful in determining the critical micelle concentration (*cmc*) of *HTAB* in *PBS* (sodium phosphate buffer—10 mM, NaCl—0.154 M pH 7.4) at 37 °C. Thus, the emission spectra of pyrene for different *HTAB* concentrations in the abovementioned conditions were recorded on a Jasco FP-6300 spectrofluorometer also equipped with a thermostating module (operating parameters: band width—5 nm; data pitch—0.5 nm; scanning speed—100 nm/min; spectrum accumulation—3; path length—10 mm using a Quartz SUPRASIL cell).

Due to *HTAB*-based composition of the release environment, all absorption spectra were acquired by using a solution of 3 × 10^−4^ M *HTAB* in sodium phosphate buffer *PBS* (*PBS-HTAB*, pH 7.4) at 37 °C as reference.

#### 2.3.4. Buffer Uptake into the Collagen-Based Matrices

Buffer (*PBS*-*HTAB*) uptake measurements were performed at 37 °C and the results were expressed as the ratios between the mass of the swollen-in buffer samples and the initial mass of the corresponding freeze-dried matrices (g/g). The weighing of the swollen samples was performed every 5 min within the first 30 min, then every 15 min for 4–5 h. The final weighing was made after 24 h, which practically corresponded to the equilibrium swelling of the samples. Three replicas were used for each of the collagen-based matrices investigated. The data were obtained as mean values ± relative standard deviation (RSD). RSD values were less than 15% for all the experimental data.

#### 2.3.5. Resistance to Collagenase Digestion

The time course of enzymatic degradation of collagen was followed using collagenase type I (from *Clostridium histolyticum*) in physiological conditions: *PBS-HTAB* of pH 7.4 at 37 °C. Pieces of 1 × 1 cm porous collagen-based matrices immersed in 1mg/mL collagenase solution were monitored for 9 days. The aspect of solutions and matrix pieces were noticed through visual inspection.

#### 2.3.6. In Vitro Release of TA and CHDG

In vitro release of *TA* and *CHDG* contained within the collagen-based matrices was performed by employing USP 2 equipment (paddle apparatus). A cylinder-shaped sample (porous matrix) was immobilized at ca. 3–4 mm above the vessel base. To ensure that the swelling of matrix and releasing of *TA*/*CHDG* occurred only by the lateral surface of the sample as a disc-shaped slice (cylindrical shape), both the bottom and upper faces of the matrix were covered by a thin paraffin wax layer (resulting from solvent evaporation of a proper solution of paraffin in petroleum ether). The release medium consisted of 1 L of 3 × 10^−4^ M *HTAB* in *PBS* (*PBS*-*HTAB*, pH 7.4) at 37 °C and a stirring speed of 50 rpm. The fraction of *TA*/*CHDG* release was calculated with respect to their amount initially contained within the dry collagen-based matrices. Measurements (performed over a time scale of 11 h) at each time point took place in triplicate and mean and RSD values were calculated. RSD values for all these experimental data were found to be less than 10%.

## 3. Results and Discussion

### 3.1. FT-IR Spectroscopy Analysis of Freeze-Dried Collagen-Based Matrices

**Figure 1:**
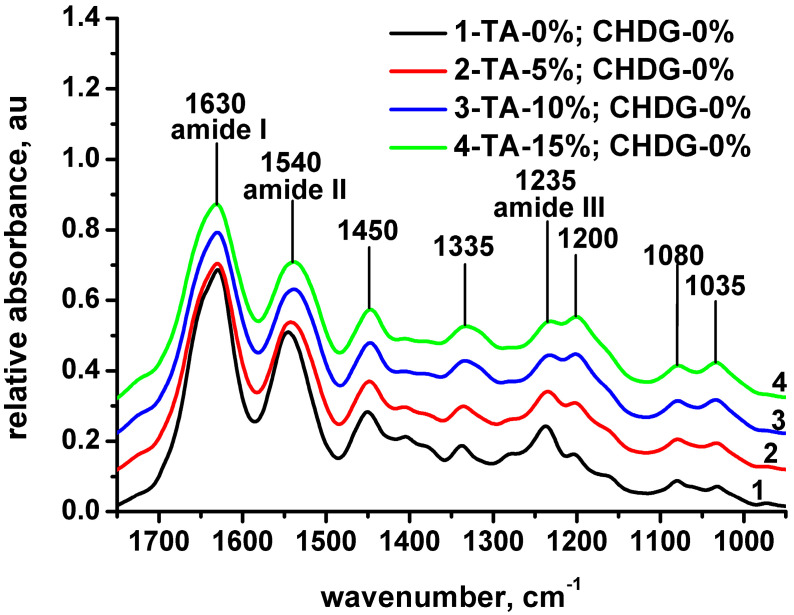
FT−IR spectra of the collagen and collagen-TA matrices with the specified compositions.

The IR absorption bands typically considered to be specific to the peptide functional groups in collagen are amide A (3330–3325 cm^−1^), amide B (3080 cm^−1^), amide I (1650 cm^−1^), and amide II (1550 cm^−1^) [59,60]. Many other published results [61,62,63,64,65,66] take into account the amide III band in characterizing the collagen microstructure. FT-IR spectra discussed below concern the matrices of collagen alone on the one hand, and four series of three collagen-based samples (one series of 5, 10, and 15% *TA* with no *CHDG* and three series containing 5, 10, and 15% *TA*, each of them with the same content of *CHDG*—1.82, 4.55, and 9.09%) on the other hand (Figure 1 and Figures S1–S3). The assignment data for all the samples investigated are collected in Table 1 and Table S1 (in Supplementary Materials).

The main IR absorption bands associated with the polypeptidic structure of collagen are clearly revealed in its FT-IR spectrum (Figure 1 and Figure S1a). Thus, the amide A band is centered at 3301 cm^−1^ and is considered to be arisen from NH stretching vibrations. This is a valuable indicator of the presence of hydrogen bonds between the collagen chains in the matrix because the free NH vibrations generally take place at 3400–3440 cm^−1^ and the band position experiences a red shifting when the NH part of a peptide linkage is involved in hydrogen bonding [60,61,62,63,64,67,68,69,70]. Even though the amide I band is sensitive to the secondary structure of a polypeptide [62,63,71,72], much more information can be obtained taking into account the amide I, II, and III bands together. In this respect, preserving the integrity of collagen triple helix may be empirically confirmed by considering either the difference in wavenumbers of the amide I and amide II bands (*ν_amideI_*-*ν_amideII_*) [73], that has to be lower than 100 cm^−1^, or by the intensity ratio of the amide III band to that located at 1450 cm^−1^ (*A_amideIII_/A_1450_*), that has to be close to unity [61,62,74,75,76]. Accordingly, IR spectra of all collagen-based matrices indicate a nondenatured, native state of collagen: *ν_amideI_-ν_amideII_* = 85–95 cm^−1^ and *A_amideIII_/A_1450_* = 0.9 (see Table 1).

Concerning the collagen-*TA* matrices, due to *TA* addition, the amide A band shifted to lower wavenumbers, suggesting an enhancement of hydrogen bonding most likely between collagen (NH of peptide groups) and *TA* (hydroxyl phenolic groups) (Table 1, Figure S1a). A similar red-shifting tendency is obeyed by the amide II and amide III bands (Table 1, Figure 1), which supports the idea of increasing hydrogen bonding between collagen and *TA* in these matrices. Moreover, some peculiar features in the IR spectra of collagen have been identified with increasing *TA* content. Thus, the absorption band centered at ca. 1335 cm^−1^, ascribed to in plane OH bending [77] (associated with tyrosine residues), ascends in its relative intensity and broadness as *TA* concentration increases.

**Figure 2:**
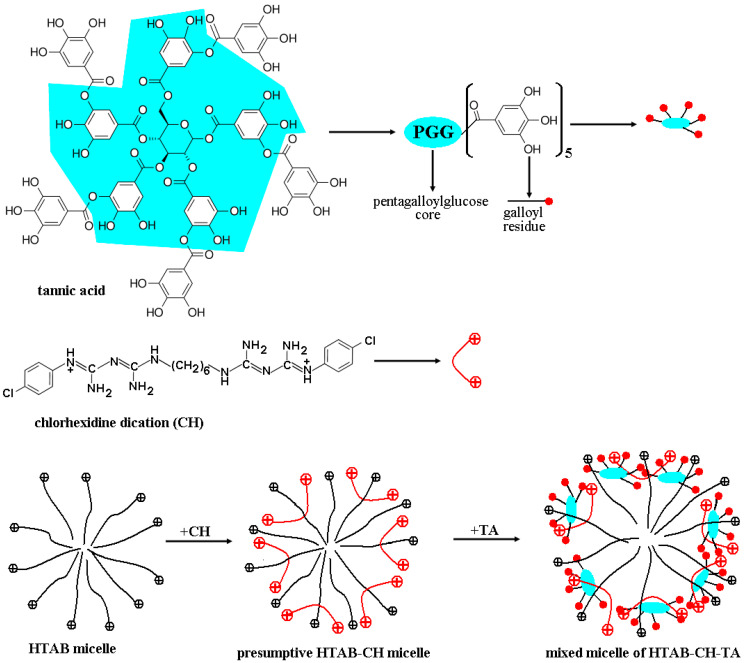
Mixed micelle formation in *HTAB* solution based on the structural characteristics of *TA* and *CHDG* (see the text for explanations).

This is reflected by the evolution of the *A_amideIII_/A_1335_* ratio from 1.3 to 1.1 when the *TA* amount varies from 0 to 15% and arises from the corresponding OH phenolic groups (some of them belonging to the pentagalloylglucose core and the others carried by the five galloyl residues within the outer zone of each *TA* molecule, Figure 2), occurring in the samples. Similarly, the relative intensity of the band located at about 1200 cm^−1^ assigned to C-O stretching vibrations (C-OH in phenol) [73] follows the same ascendant course as *TA* concentration increases (*A_amideIII_/A_1200_* is 1.48 for collagen and 0.95 for collagen with 15% *TA*). At the same time, the IR bands centered at 1080 and 1035 cm^−1^ assigned to C-O stretching vibrations of carbohydrate moieties [64,72,78] enhance in their relative intensities when *TA* content rises. This is due to the progressively increasing contribution of the ester groups in *TA* to IR signals at those wavenumbers (C-O-C symmetrical stretching) that overlap the specific bands of carbohydrate moieties bound to collagen.

To assess the simultaneous presence effect of *CHDG*–*TA* (at the maximum content of *CHDG*) on the secondary structure of collagen and the hydrogen bonding in *CHDG*–*TA*–collagen systems, a series of three matrices with the 9.09% *CHDG* and various *TA* concentrations (5, 10, 15%) were subjected to FT-IR investigations. Surprisingly, neither amide A band, nor amide II and amide III bands indicate supplementary enhancement in hydrogen bonding between collagen molecules themselves and collagen–*TA*/*CHDG*s. On the contrary, the blue-shifting in the amide A band for the *CHDG–TA*–collagen-based matrices with 5 and 10% *TA* confirm a little diminishment in collagen–collagen hydrogen bonding (Table 1). This does not exclude the possible interactions through hydrogen bonding between collagen and *TA*/*CHDG* that may imply types of collagen functional groups other than peptide linkages: *−COOH* of glutamic and aspartic acid residues, *−OH* of tyrosine, serine, and threonine. These hydrogen bonds may also be accompanied by hydrophobic interactions mainly established between galloyl residues of *TA* and the hexamethylene bridge of *CHDG* on the one hand, and these parts of *TA*/*CDDG* molecules and some hydrophobic regions of collagen [18,19]. Therefore, such combined hydrogen bond/hydrophobic interactions could play a significant role in the physical crosslinking of collagen in the matrices. Otherwise, the same picture regarding the changes in IR patterns of collagen–*TA* matrices (at ca. 1335, 1200, 1080, and 1035 cm^−1^) was also found for the collagen–*TA*–*CHDG* samples (Figure S1). However, due to the specific vibrations of guanidine groups of *CHDG* [68,79] (C=N symmetrical stretchings at ca. 1630 cm^−1^ overlapped by the amide I band and C−N stretching at 1235 cm^−1^ overlapped by the amide III band of collagen), the relative intensities taken as the *A_amideIII_/A_1200_* ratio were found to vary from 1.48 for the collagen alone sample to 1.01 for the *TA*–*CHDG*–collagen with 15% *TA*. FT-IR spectra for the rest of the collagen–*TA*–*CHDG* matrices exhibit very similar features as those discussed above and can be readily seen in Table S1 and Figures S1–S3.

### 3.2. SEM Investigations of Matrices Morphologies

**Figure 3:**
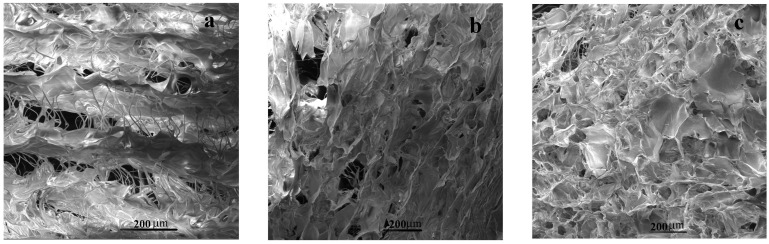
SEM micrographs (taken from cross-section) for (**a**) collagen matrix and collagen-based matrices with (**b**) *TA* (10%) and with (**c**) *TA* (10%)-*CHDG* (9.09%).

As pointed out previously [5], the porous structure of collagen and collagen-based matrices is a direct result of a negative replica formation of the ice network (in this case the dendritic crystals of aqueous acetic acid solution in the frozen state) generated during the first stage of lyophilization, followed, eventually, by the ice sublimation at a low pressure and temperature set adequately to the freeze-drying process. In this respect, by controlling the freezing step of the initial collagen-based solutions/suspensions, a large variety of pore morphologies could be obtained for the final systems. Thus, it was experimentally established that the lower the temperature of freezing, the smaller the pore size, and, depending on the quality of heat transfer in the course of lyophilization, the pore orientation may be altered [5]. Even though all the matrices were obtained simultaneously during the same freeze-drying procedure, SEM investigations revealed some differences in porous morphology according to the systems’ composition. For the sponges consisting of collagen solely, a well-defined lattice-like lamellar morphology was observed in a cross-section taken perpendicular to the air-sided plan (Figure 3a), whereas a network of rather regular open pores (of ca. 200 μm in diameter, on average) was detected when exploring sections parallel to the air side of the matrix (Figure S4). Such morphologies are fairly common and have been also reported [5,80,81] for collagen/collagen-based matrices obtained via freeze-drying. Instead, the presence of *TA* at concentrations of 10 and 15% brings some disorder degree both in lamellar and pore orientation (see Figure 3b and Figure S4b) of the collagen–*TA* sponges. However, this feature of “random” pore orientation is actually an advantage of using this type of matrix for soft tissue (such as skin) regeneration purposes [5]. At the same time, the pore size distribution seems to be far broader in these collagen–*TA* systems compared to the collagen matrices (except for the collagen–*TA* (5%) mixtures that look like similar, morphologically speaking, to the collagen ones). By introducing the other low molecular weight compound (*CHDG*), the morphology of the three-component matrices has progressively changed in the sense that the characteristic lattice-like lamellar structure and pore orientation have been partially lost with an increasing the *CHDG* percentage. While the systems of collagen–*TA* (5%)–*CHDG* (1.82%) resemble the collagen and collagen–*TA* (5%) matrices to a great extent, all the other three-component freeze-dried mixtures exhibited quite different morphologies. Particularly, the higher the concentrations of *TA* and *CHDG*, the larger the disorder in the lamellar structure and pore orientation. Moreover, a high content of *TA* and *CHDG* partially induced the pore collapse (Figure S4c), in addition to a visible disorder in the lattice-like lamellar morphology (Figure 3c) of these three-component sponges. Practically, the most affected matrices from this point of view were the collagen–*TA* (15%)–*CHDG* (9.09%) ones. On the other hand, all the matrices investigated revealed a porous aspect ion the surface in contact with the molding support and a relatively smooth air-sided face as a result of pore collapse during freeze-drying (images not shown). These peculiarities were also experimentally demonstrated more than three decades ago [80,82] and, based on that, the reason for why the pan side of a lyophilized collagen matrix should be brought, for example, into direct contact with the skin, particularly when such kind of sponge is intended for wound dressing [80].

### 3.3. Isotropic Swelling of Collagen-Based Matrices in an Aqueous PBS-HTAB Environment

The remarkable capacity of collagen matrices to retain aqueous medium is due to the numerous hydrophilic moieties of collagen molecules/fibrils, such as amide, amine, carboxyl, and hydroxyl functional groups, as is the case with most polar polymers [83]. Its swelling behavior in aqueous media is very important for the use of collagen matrices as wound dressings, firstly in ensuring the hydration of the tissue surface on which the matrix is applied and the releasing of the drug contained by the matrix placed onto the wound surface. Moreover, swelling with biological fluids is the main compulsory step in the process of matrix degradation.

**Figure 4:**
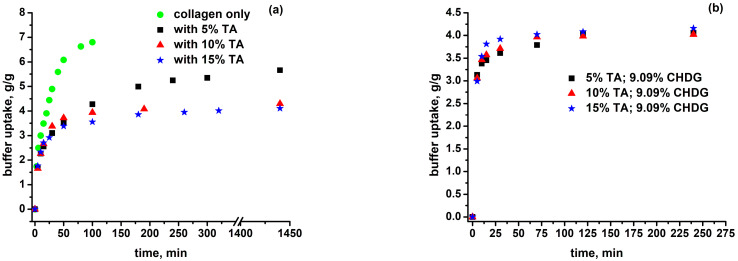
*PBS–HTAB* uptake into the matrices containing (**a**) *TA* and (**b**) *TA–CHDG* at the specified compositions and 37 °C.

The swelling measurements were carried out in a *PBS–HTAB* solution on freeze-dried matrices of collagen solely, collagen with *TA* (5, 10, 15%), and collagen with *TA* (5, 10, 15%) and *CHDG* at its maximum content used (9.09%), obtained from the corresponding acidic hydrogels. The reason why the *PBS-HTAB* solution was chosen as the swelling solvent is rationally explained later in the section dedicated to *TA/CHDG* release. The experimental results are graphically shown in Figure 4 and the plotted values were obtained after a correction of the raw data with the amount of *PBS–HTAB* solution physically retained (by capillarity) into the micrometer-sized pores of the dry matrices. To do that, each porous collagen-based sample intended to be swollen in the *PBS–HTAB* solution was firstly soaked in anhydrous ethanol (where matrices morphology and volume are practically not affected) in a pycnometer at 20 °C (at which the pycnometer was calibrated) to completely fill the pores. The volume of ethanol retained by the sample (calculated from the measured weight of filling ethanol and its density) was considered to be the same as that of the aqueous solution of *PBS–HTAB*. This was the first step in assessing the fraction of *PBS*–*HTAB* uptake in a matrix corresponding to mechanically filling its pores from the total aqueous solvent retained by the sample during its swelling. In fact, the real swelling data resulted from removing the capillary contribution to reflect the capacity of solvent retaining exhibited by a matrix based only on the hydrophilicity of the sample components and degree of crosslinking. More than that, due to the fact that the swelling measurements were performed at 37 °C by weighing, the evaluation of the capillary contribution to the process and the degree of swelling expressed on the basis of volume fractions (useful in the next section) required the most reliable value of the density of the *PBS–HTAB* solution at that temperature. Therefore, starting from a well-known change in water density over a given temperature range, and considering the same course of tendency for the temperature dependence of *PBS–HTAB* density, a density value of 1.093 g/cm^3^ at 37 °C was estimated for the buffer solution after an initial experimentally determined value of 1.098 g/cm^3^ for the same solution at 20 °C (for specific details, see Figure S5).

Referring back to the data in Figure 4, the collagen matrices displayed the highest solvent uptake (6.80 g/g at 100 min), but they became progressively more gelatinous and, after ca. 120 min, broke apart up into tiny gelatinous pieces dispersed into the aqueous environment. Alternatively, the collagen-based matrices with 5% *TA* showed a much lower swelling degree. Additionally, the samples retained their physical integrity, becoming gelatinous after a 24 h immersion (buffer uptake at 24 h was 5.66 g/g). This behavior is a direct consequence of collagen crosslinking via *TA* molecules, especially through hydrogen bonding and even hydrophobic interactions. Increasing *TA* content to 10% resulted in a significantly lower buffer uptake near to equilibrium (4.30 g/g at 24 h), although the swelling rate was rather similar to that of matrices with 5% *TA* at the onset of the swelling process (within the first 50 min). Going to 15% *TA* content, the matrices exhibited almost the same swelling behavior as the samples with 10% *TA*, with a buffer uptake of 4.11 g/g after 24 h of immersion. The last results support the supposition of a saturated crosslinking of collagen by *TA*, beyond which the swelling capacity in the *PBS–HTAB* solution does not change significantly. As partially discussed in the previous section on FT-IR data, *TA* entities may build up crosslinkages through H-bonding within collagen matrices involving –*OH* groups linked to the pentagalloyl core and galloyl residues of *TA* (see Figure 2), and potentially some specific moieties of collagen: side –*OH* groups of serine and hydroxyproline residues, side –*COOH* or –*COO^−^* groups of aspartic and glutamic acid residues, amine groups of lysine residues, or amide groups of asparagine and glutamine residues [17]. Every single functional group involved may play the role of either hydrogen bond donor or hydrogen bond acceptor. At the same time, the aromatic rings of the galloyl groups of *TA* structure are considered to be responsible for strong hydrophobic interactions, with nonpolar regions of collagen molecules [18,19] having a direct consequence in an increased hydrothermal stability of *TA*-treated collagen compared to the *TA*-free collagen. Thus, computational docking carried out on the *TA*–collagen-like polypeptide (*CLP*) system suggested formation of hydrophobic “bonds” between the aromatic rings of *TA* and glycine, methionine, glutamic acid, serine, arginine, and proline residues on *CLP*, and a number of the hydrogen bonds between *TA* and *CLP* [18].

On the other hand, and quite unexpectedly, all the matrices loaded with *TA* and with the maximum content of *CHDG* (9.09%) have shown buffer uptake, with time traces very close to each other irrespective of *TA* relative amount. At the same time, the values of swelling degrees were found to be 4.16, 4.02, and 4.06 g/g for the *TA–CHDG*–collagen sponges with *TA* content of 15, 10, and 5%, respectively, which are practically the same as for that of collagen-based samples loaded with 15% *TA* (for comparative purposes only, where the capillary contribution dominates, the raw data for the maximum swelling ratios ranged from 111.32 g/g for the collagen matrices, to 92.62 g/g for the most swollen *TA*-collagen sponges, and to about 68 g/g for the three-component matrices with the maximum content of *CHDG*). Such behavior proves the existence of the supplementary attractive interactions brought by *CHDG* over the entire volume of the three-component matrix when the porous collagen network contains lower proportions of *TA* (5 and 10%). In other words, a saturation degree of crosslinking has been reached for these collagen-based matrices at this content of *CHD*G (9.09%). In fact, the effect of the chlorhexidine species endowed with so strong a crosslinking ability onto the swelling behavior of the studied porous collagen-based systems could be mainly attributed to two categories of attractive interactions:

- Electrostatic interactions between the chlorhexidine dications (*CH^++^*) and the collagen entities negatively charged in the swelling solvent (for more details, see the next section), with a substantial enhancement in the general crosslinking of the collagen network, and
- So-called Yoshida forces or Yoshida H-bonding [84], as quite strong attractive interactions established between proton-donating groups/molecules and π-electron bases represented by entities with delocalized π-electron clouds, especially aromatics.

Strong sorption of a 4-vinylpyridine-based polymer partially quaternized or of chlorhexidine (*CH*) on cellulose are two classical examples of how Yoshida forces (between an electron-deficient hydrogen linked to an oxygen atom of an *–OH* group anchored onto a β-D-glucose residue of cellulose and π electrons belonging to aromatic ring of pyridine/pyridinium [85] and to p-chlorophenyl moieties of *CH* [86]) act. Adapting this to our collagen-based systems, there are many electron-deficient hydrogens linked to the collagen structure (of peptide bonds, of *–OH* groups contained in hydroxyproline residues, tyrosine, serine, threonine, of unprotonated primary amine groups of lysine residues) able to interact with π electrons belonging to the aromatic rings of *CH* via Yoshida H-bonding, leading to increasing of the crosslinking degree of the collagen network. On the other hand, the favorable interactions between *CH* and *TA* through the same Yoshida forces, and even electrostatic ones, together with the contribution of the hydrophobic effect caused by the presence of aromatic rings, make this situation more and more complex, which is why it deserves proper attention in further investigations.

### 3.4. Degree of Crosslinking Estimation for Collagen-Based Matrices Revealed by Equilibrium Swelling Measurements in an Aqueous Environment

To estimate the degree of crosslinking (*DC*, in mol∙cm^−3^) associated with the collagen-based matrices, a useful relationship developed by Flory [87] for polyelectrolyte gels at equilibrium swelling could have been applied:

(1) $$DC=\frac{V_{1}\cdot \Delta c-[ln(1-\nu_{2eq})+\nu_{2eq}+\chi_{1}\cdot \nu_{2eq}^{2}]}{V_{1}\cdot (\nu_{2eq}^{1/3}-\nu_{2eq}/2)}$$

where *V*_1_ is the molar volume of the solvent, Δ*c* = *c* − *c_i_*—the difference in the total concentration of ionic mobile species (in mol∙cm^−3^) found inside (*c*) and outside (*c_e_*) the swollen gel, *ν_2eq_*—the volume fraction of macromolecular component in the gel at equilibrium swelling, and *χ*_1_—the Flory–Huggins interaction (polymer–solvent) parameter. However, Equation (1) is mainly applicable to those polyelectrolyte gels crosslinked in a bulk state prior to swelling. For a gelation process occurring in solution at a certain volume fraction of a nonionic polymer, *ν*_20_, Bry and Merrill [88] have derived a corresponding relationship that has then been adapted to a polyelectrolyte network at the same state of equilibrium swelling by Ofner and Bubnis [89]:

(2) $$DC=\frac{V_{1}\cdot \Delta c-[ln(1-\nu_{2eq})+\nu_{2eq}+\chi_{1}\cdot \nu_{2eq}^{2}]}{V_{1}\cdot \nu_{20}\cdot [{\left(\nu_{2eq}/\nu_{20}\right)}^{1/3}-(\nu_{2eq}/2\nu_{20})]}$$

Given the nature of the solvent (aqueous environment) in Equation (2), the quantity *V_1_* is the molar volume of water and the dimensionless quantity *χ*_1_—the Flory–Huggins interaction parameter of the collagen–water pair was considered to be 0.49. This value was chosen in accordance with the results published elsewhere [90] for gelatin and based on the finding that both gelatin and collagen have the same *χ*_1_ value at high relative humidity (>95%) [91] and, most likely, even in aqueous systems.

For all the systems discussed herein, the overall difference in the mobile ion concentration inside and outside the matrix was evaluated by considering the Donnan equilibrium [92] established between the releasing environment (phosphate-buffered saline, *PBS*) and the polyelectrolyte network at pH 7.40 and 37 °C. Thus, according to the net electrical charge of collagen, the distribution of the mobile ions inside and outside the matrix at equilibrium has to fulfill the condition of equality between the activities of the electrolyte existing in the two zones of the swollen systems. Prior to discussing the consequences of the Donnan effect in these systems at equilibrium swelling, some prerequisites were assumed:

- Calculation of the total ionic strength corresponding to the solution of *PBS* (10 mM phosphate buffer, 154 mM NaCl) at pH 7.40 and 37 °C;
- Writing the equation of the Donnan equilibrium by considering an amount of NaCl equivalent to the total ionic strength produced by *PBS* at the operational parameters.

**Figure 5:**
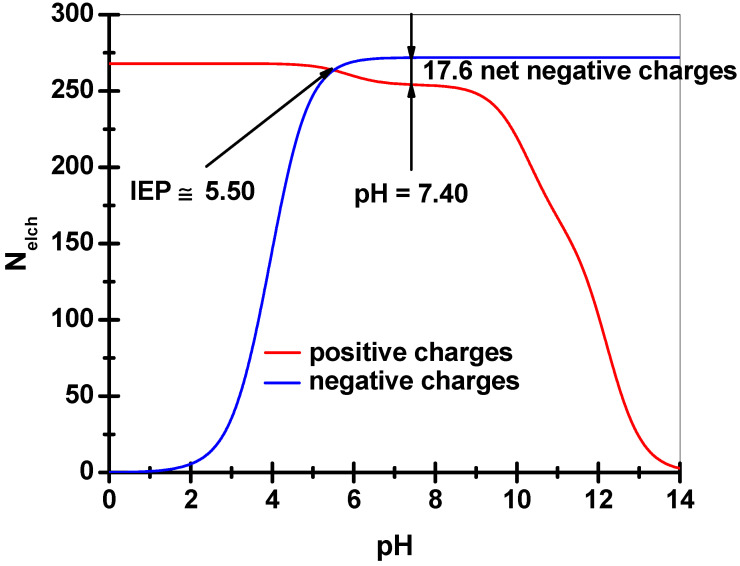
Number of fixed electrical charges (*N_elch_*) associated to one collagen type I molecule (atelocollagen) on pH changing (adjusted to the operating conditions: pH = 7.40, *I* = 0.180, 37 °C).

Based on the abovementioned conditions, the ionic strength corresponding only to the concentration of 10 mM phosphate buffer in the presence of 154 mM NaCl (considered fully dissociated) was found to be 0.026 (see Supplementary Materials). Thus, the total ionic strength (*I*) is 0.180, the equivalent concentration of NaCl leading to this *I* value is 0.180 M, and, going further, half the overall concentration of the mobile ionic species (NaCl was considered fully dissociated), *c_e_*, is 0.090 M. Therefore, according to what the Donnan effect mainly means [92] (the fulfillment of the equality condition between the activity of the mobile electrolyte inside and outside the matrix—which is expressed as an equality between the products of the constituent ion activities of the mobile electrolyte—and also of the electroneutrality condition involving all ionic species into the two regions), the following equation will finally result:

(3) $$\Delta c={\left(c_{p}^{2}+4c_{e}^{2}\right)}^{1/2}-2c_{e}$$

where *c_p_* is the concentration of net fixed electrical charge of collagen in the swollen matrix (ionic strength of 0.180, pH 7.40, and 37 °C). In deriving Equation (3), the mean ionic activity coefficient of the electrolyte (see details in Supplementary Materials) was considered to be the same both inside and outside the matrix. To estimate the *c_p_* value, it is necessary to evaluate the number of net fixed electrical charges (*N_elch_*) onto every single collagen molecule (atelocollagen). To do so, based on the amino acid composition, the residues of aspartic acid (*Asp*), glutamic acid (*Glu*), histidine (*His*), arginine (*Arg*), and lysine (*Lys*) have been taken under consideration in generating the fixed charges attached to the collagen type I triple helix depending on pH, temperature, and the ionic strength of the swelling environment (see Table S2). For simplicity, the following conjugate acid/base pairs were taken into account: *–COOH/–COO^−^* pairs for the residues of *Asp* and *Glu* and *–NH_3_^+^/–NH_2_* pairs for *His*, *Arg*, and *Lys* residues. Their acidity exponents (*pK_a_*) at 25 °C and with no electrolyte (Table S2) were corrected according to the influences induced by the temperature and ionic strength changes (*t* = 37 °C, *I* = 0.180) [4,93,94,95,96,97]. The corrected *pK_a_* values (Table S2, recorded in parentheses) were obtained by applying equation (S13), where the quantity *Z* is −1 for the *–COOH/–COO^−^* pairs and +1 for the *–NH_3_^+^/–NH_2_* pairs as a result of a comparison to the equilibrium (S2) (in Supplementary Materials). To account for all the presumable electrical charges per one triple helix of collagen type I as atelocollagen (without terminal telopeptides cleaved enzymatically), the corresponding number of *N*-terminal and *C*-terminal amino acid residues (three glycine residues for *N*-terminus and three proline residues for *C*-terminus) were also considered (Table S2). This is in accordance with the primary structure of the three polypeptide chains belonging to the triple helical structure of the bovine collagen type I [98]. Starting from the well-known Henderson–Hasselbalch equation applied to all the conjugate acid/base pairs discussed above, the number of net electrical charges carried by a molecule of atelocollagen (*N_net_*) at 37 °C, pH 7.40, and *I* 0.180 will be given as:

(4) $$N_{net}=\sum_{i}N_{i}\cdot \frac{10^{pH-{\left(pK_{a}\right)}_{i}}}{1+10^{pH-{\left(pK_{a}\right)}_{i}}}-\sum_{i}N_{j}\cdot \frac{1}{1+10^{pH-{\left(pK_{a}\right)}_{j}}}$$

where the first term of the right side of the equation denotes the overall number of the negative charges and the second, the total number of the positive charges. The subscripts *i* refer to each of the identical amino acid residue types carrying carboxylic groups in the protonated form (*Asp*, *Glu*, and *Pro*, respectively) and *j*, a similar quantity defined for the identical amino acid residues types bearing basic groups in the deprotonated form (*His*, *Arg*, *Lys*, and *Gly*, respectively). Therefore, *N_i_* and *N_j_* are the number of identical amino acid residues belonging to each of the residue kinds just mentioned (*Asp*-90, *Glu*-150, *Pro*-3, *His*-14, *Arg*-162, *Lys*-89, and *Gly*-3, respectively) and *(pK_a_)_i_* and *(pK_a_)_j_* are the corresponding acidity exponents (see Table S2). Because the isoelectric region of collagen was found to be 5.0–6.0 (determined from the maximum optical density of 0.03% collagen in 0.180 M NaCl aqueous solution at different pHs, results not shown), a value of 5.5 has been chosen for its isoelectric point (*IEP*), where *N_net_* becomes zero, and, as a consequence, the number of fixed negative charges should be greater than that of the fixed positive charges at pH 7.40. Instead, the calculated *IEP*, by counting the contributions of the overall fixed charges, was found to be higher than 8.0, a value far greater than that experimentally determined. A possibility of somehow matching the two very different *IEP* values is to consider a larger number of acid amino acid residues (*Asp*, *Glu*) actually carried by the collagen type I molecules used in this study. This is truly feasible if the asparagine (*Asn*) and glutamine (*Gln*) residues undergo a deamidation to a certain extent to give additional carboxylic groups. Indeed, by taking into account a degree of deamidation of 24% and keeping the same ratio of *Asn/Gln* (Table S2), a supplementary number of *Asp* (11) and *Glu* (18) could result, and, consequently, an *IEP* value of 5.5 was calculated as shown in Figure 5 (by using Equation (4)). Such an acidic *IEP* of collagen could be attained due to the extraction procedures of collagen partially conducted at quite basic pH when some side carboxamide groups of *Asn* and *Gln* might be easily hydrolyzed to the corresponding carboxylic groups. Once a very good accordance between the experimental and calculated values of the collagen *IEP* was established, the next step was to estimate the number of net negative charges carried by a molecule of atelocollagen at pH 7.40, *I* 0.180, and 37 °C. As can been seen in Figure 5, this quantity may be easily obtained based on Equation (4), giving a value of 17.6 and, going further (by taking into account the expressions (S17)–(S20) in Supplementary Materials), the *c_p_* values (in mol/cm^3^) will be given by:

(5) $$c_{p}=17.6\cdot \frac{\rho_{coll}}{Q_{v,\mathrm{coll}}^{\mathrm{eq}}\cdot M}=17.6\cdot \frac{\nu_{2\mathrm{eq}}\cdot \rho_{\mathrm{coll}}}{M}$$

where $Q_{v,\mathrm{coll}}^{\mathrm{eq}}$ is the equilibrium volume swelling ratio regarding only the collagen component (with density *ρ_coll_*, in g/cm^3^) of a given collagen-based matrix of density *ρ_matrix_* (in g/cm^3^) and *M*—the molar mass of collagen (considered 3 × 10^5^ g∙mol^−1^). The values of the quantities preceding c_p_, as well as the c_p_ values themselves, and the Δc values, are listed in Table 2 and Table 3 for all the studied matrices. Eventually, with these intermediate steps accomplished, it was possible to calculate the crosslinking degrees of collagen-based matrices (by Equation (2)) supposing that *ν_20_* is on average 0.20. This value was chosen in accordance with the percentage of moisture content found in the fresh hide/skin of mammals (ca. 70%) and the percentage content of collagen in dry skin (ca. 75%) [98,99,100,101,102,103].

Due to the fact that the real *ν_20_* values (the collagen volume fraction in a certain swollen collagen-based matrix at equilibrium) are difficult to find, there are two limiting cases where this problem can be readily solved (by keeping in mind the same matrices densities that were picnometrically determined): the first of them, when neither *TA* nor *CHDG* were released at equilibrium swelling (Table 2), and the second, when practically both *TA* and *CHDG* were considered almost majority released at equilibrium (Table 3). Accordingly, the degrees of crosslinking (*DCs*) corresponding to the two limiting cases grew higher from 58 × 10^−5^ mol⋅cm^−3^ for collagen matrices to (86–103) × 10^−5^ mol⋅cm^−3^ for collagen–*TA* (5%) sponges, maintaining this tendency as *TA* content increased to 15% ((191–306) × 10^−5^ mol⋅cm^−3^). On the other hand, *DCs* varied from (208–321) × 10^−5^ mol⋅cm^−3^ to (184–336) × 10^−5^ mol·cm^−3^ and further to (144–297) × 10^−5^ mol⋅cm^−3^ for the three-component porous matrices with the same content of *CHDG* (9.09%) and *TA* percentage rising from 5 to 15%, respectively (Table 2 and Table 3). The *DC* values are, most likely, closer to those specific to the swollen collagen-based sponges with the two low molecular weight components (*TA* and *CHDG*) almost entirely released. This is because the fractional release after 660 min (denoted as c_11_ in Supplementary Materials) ranges from 0.7 to 0.9 for *TA* and ca. 0.4–0.6 for *CHDG* depending on the matrix composition, as can be seen later in the Section 3.6 (see also Figure S6). Anyway, irrespective of the limiting cases mentioned above, the ascendant tendency of *DC* values follows exactly the decrease of $Q_{v,\mathrm{coll}}^{\mathrm{eq}}$ values for all the matrices investigated (Table 2 and Table 3, the 4th and 8th columns). Unlike the sponges made of collagen solely, which are practically non-crosslinked (*DC* value of 58 × 10^−5^ mol⋅cm^−3^ corresponding to a swelling time of 100 min is purely informative because these kinds of matrices began to disintegrate into smaller and smaller pieces as time passed, see Figure 5), the collagen–*TA* matrices behaved completely differently, showing the key role played by *TA* in making physical crosslinkages between the collagen molecules. Thus, a gradually increasing percentage of *TA* led to enhancing in *DC*s of lyophilized collagen–*TA* sponges, as arisen from the decreasing values of equilibrium of the weight/volume swelling ratio for the same bicomponent systems (Figure 5 and Table 2 and Table 3). On the other hand, the presence of *CHDG* at its maximum percentage (9.09%) somehow impairs the physical crosslinking of collagen in matrices with *TA* and *CHDG* when *TA* content rises (see the values of $Q_{v,\mathrm{coll}}^{\mathrm{eq}}$ and *DC* in Table 2 and Table 3). Taking into account the more realistic case of about total release of *TA* and *CHDG* from the three-component systems (containing the same proportion of *CHDG* of 9.09%) at equilibrium swelling (Table 3), both $Q_{v,\mathrm{coll}}^{\mathrm{eq}}$ and *DC* values are very close to those associated with *TA* (15%)–collagen matrices. These data support the idea of reaching the maximum value of the *DC* of collagen network for all these systems (by the crosslinking contribution brought by *TA* and *CHDG* taken together or separately), and, in addition, are consistent with the experimental findings discussed earlier concerning the similarities noticed in swelling behavior. Other specific details on building up the physical crosslinkages in the collagen-based matrices studied are discussed later in the section regarding *TA/CHDG* releasing.

### 3.5. Collagenase Digestion

The collagen type I (which is the main component in all the samples studied) in its native state resists the attack of most proteolytic enzymes, except for the specific collagenases. These so-called matrix metalloproteinases are able to cleave through each individual triple helix at a point approximately three-quarters of the distance from the *N*-terminal end along the collagen molecule (usually at a peptide bond between *Gly* and *Leu* or *Gly* and *Ile* amino acid residues). This pathway of enzymatic digestion was also described for the collagen type II and III [4,104,105,106,107]. Degradation of collagen fibrils starts from the outside: the enzyme fastens tightly onto molecules from the surface, and the inside ones are accessible by progressive degradation [4,107]. Once the triple helix cleaved, the fragments remain attached to the fibril (in the case of collagen fibrils) by crosslinks occurring in mature collagen (fibrils), and, at body temperature (ca. 37 °C), they start to denature [104]. At the same time, gelatinases or other nonspecific proteinases continue the degradation, digesting the primary fragments into low molecular weight peptides and eventually amino acids [4]. Due to the specific enzymatic action only onto triple helices taken individually, collagenases are frequently used for in vitro collagen digestion.

The collagen-based matrices studied in this paper were subjected to digestion by using collagenase type I (from *Clostridium histolyticum*) in a *PBS*–*HTAB* environment at pH 7.4 and 37 °C.

The matrices consisting of fibrillar collagen solely disappeared within ca. 30 min and the digestion aqueous medium became clear. The integrity and overall shape of the matrices with 5% *TA* were kept for about 6 h, and then disintegrated into highly swollen fragments, the final aqueous system becoming a little turbid and pale green-yellowish in color.

By doubling the amount of *TA*, the collagen–*TA* matrices preserved their own integrity for 24 h, the fragments formed during the subsequent disintegration were more or less swollen, while the turbidity and color of the digestion medium intensified. By increasing the *TA* amount to 15%, the matrices resisted for 9 days (the maximum period of observation), preserving their integrity and shape, with the same appearance of the digesting medium as that of the previous samples.

The presence of both *TA* and *CHDG* into the collagen matrices increased spectacularly their resistance to collagenase: all resisted for 9 days, except those containing 1.82% *CHDG* and 5% *TA*, which, on the 9th day, showed a fragmentation into a few relatively bulky fragments. These findings indicate most likely that *TA* and *CHDG*, jointly or individually, generate physical crosslinks between collagen molecules throughout the collagen network of the studied matrices limiting the effectiveness of the collagenolytic activity of collagenase, as reported elsewhere [27]. However, beyond these noticed aspects, supplementary investigation should be taken into account to prove whether the presence of *TA* and/or *CHDG* inhibits to a greater or lesser extent the collagenase activity, as suggested Velmuragan et al. in an interesting work on *TA*–collagen type I interaction [18].

### 3.6. TA and CHDG Release in a PBS–HTAB Medium

**Figure 6:**
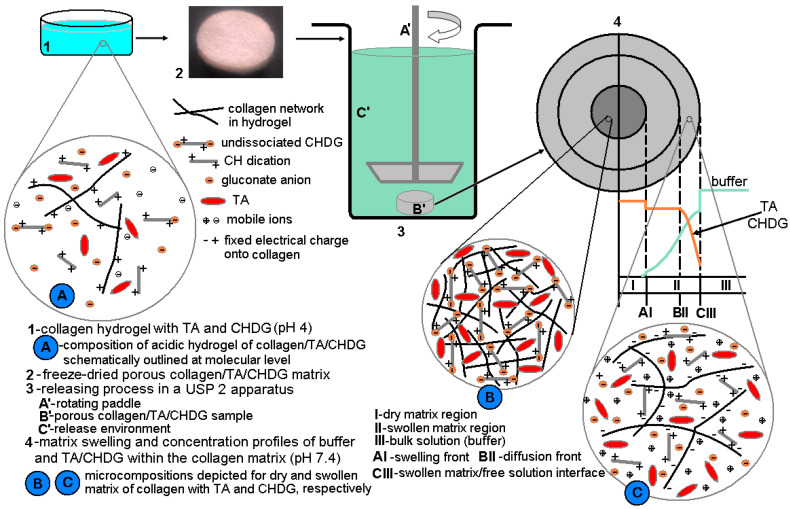
Overall scheme depicting releasing process of *TA*/*CHDG* from a collagen-based matrix prepared from an initial hydrogel (pH 4) and a subsequent step of freeze-drying.

Generally speaking, drug release from a polymeric matrix, porous or nonporous, involves a number of important aspects that may have a greater or lesser influence [108]: matrix swelling and erosion, drug dissolution, drug–matrix structure interactions, drug distribution and concentration within matrix, matrix shape (cylindrical, spherical, etc.), and matrix polydispersion. Adapting them in terms of the simultaneous release of *TA*/*CHDG* from a collagen matrix into a release environment, we took into account four main factors that could affect the releasing process, considering the same matrix shape and operating temperature: (a) *TA*/*CHDG* load (concentration) inside the matrix; (b) *TA*/*CHDG*–matrix interaction; (c) mutual *TA*–*CHDG* interactions that may yield complexes more or less soluble into the release medium; (d) *TA*/*CHDG*–release medium interactions. Based on all these features, it becomes clear that *TA*/*CHDG* in a dry collagen matrix cannot be released as solid form (micro crystals or other solid form). However, during immersion into the release fluid, the dry matrix may experience a swelling process (accompanied by a relaxation phenomenon of macromolecular network [109,110,111,112,113], followed by a corresponding *TA*/*CHDG* dissolution. Anyway, as the swelling process evolves, three moving boundaries can be defined irrespective of the swellable matrix: erosion, diffusion, and swelling front, respectively (Figure 6)) [108,113].

However, from a practical standpoint, using *PBS* (pH 7.4) at 37 °C as the release medium was a difficult task because *CHDG* could not be dissolved in that medium at all (most probably due to the chlorhexidine phosphate formation involving chlorhexidine dication, *CH^++^*, and hydrogen phosphate dianion, *HPO_4_^2^^−^*). To overcome such a drawback, we proposed a phosphate buffer with a carrier able to solubilize both *CHDG* and *TA*. Thus, we have chosen a cationic surfactant (to avoid precipitation), hexadecyltrimethylammonium bromide (*HTAB*), with low critical micelle concentration (*cmc*) [58,114,115,116,117,118,119,120,121]. This overall picture seems to be supported by the experimental findings according to which *CHDG* is soluble in pure water, NaCl solution, *HTAB* solution, or a mixed *PBS–HTAB* solution, but very insoluble in *PBS* solution. Moreover, the presupposition of insoluble *CH^++^-HPO_4_^2−^* ionic pairs formation is based on the preponderant proportion of HPO_4_^2−^ species in buffer solution at pH 7.4, compared to H_2_PO_4_^−^ ones ([HPO_4_^2−^] ≈ 1.6∙[H_2_PO_4_^−^]).

Accordingly, the release solution consisting of 0.3 mM *HTAB* in *PBS* (10 mM sodium phosphate buffer, NaCl–0.154 M, pH–7.4, which imitates human extracellular fluid from the point of view of ionic strength and pH) was employed. Such a medium was in fact a micellar solution of *HTAB*, with cationic micelles as carriers for *TA/CHDG* (mimicking somehow the function of the carrier proteins). By using pyrene as the fluorescence probe, the *cmc* of *HTAB* was found to be 7.3 × 10^−5^ M in *PBS* (pH 7.4) at 37 °C (Figure S7).

At this point, prior to discussing, in detail, *TA/CHDG* release, it is important to make some considerations on a few structural features of chlorhexidine. First of all, as a biguanide-based compound (i.e., bisbiguanide derivative), chlorhexidine is frequently represented by a misleading structure both in studies of medicinal chemistry and generally in chemistry literature. Thus, the “incorrect” structure of biguanide moiety (Table S3, 1st row) proved to be less stable than the “right” tautomer (Table S3, 2nd row) by ca. 10 kcal·mol^−1^, which resulted from ab initio calculations [122,123]. This stable structure of biguanide (asymmetric in shape and with intramolecular conjugation between C2-N3 and C4-N5 double bonds, see Table S3, 2nd row) was clearly confirmed more than 40 years ago via single crystal X-ray diffraction [124]. At the same time, the biguanide moiety is protonated in *CHDG* and, based on the calculated partial atomic charges (from natural population analysis) [123] and the absolute proton affinities [125], the most favored protonation atom to result in the biguanidinium cation is N5 (Table S3, 2nd and 3rd rows). These theoretical calculations performed on biguanide to give the structure after its protonation were also strongly supported by the crystal structure of biguanide hydrochloride revealed through X-ray diffraction [126]. Secondly, in accordance with the structure of the biguanidinium cation postulated both theoretically and experimentally, there are a number of conformers/tautomers of the chlorhexidine dication (*CH^++^*) that can adopt more or less extended conformations, as shown in Table S3 (Supplementary Materials). On the other hand, in a *PBS* environment (pH 7.4), *CHDG* ought to be most likely in double protonated form because of the *pK_a_* value evaluated to be of 9.55 for the chlorhexidine dication [86]. As already mentioned, the poorly soluble (actually, quite insoluble) complexes of *CHDG* in the releasing solution (complexes with strong ion pairing between *CH^++^* and *HPO_4_^2−^*, similar to those established between guanidinium cations and sulfate dianions [127]) will be embedded preferentially into the *HTAB* micelles. The way the chlorhexidine is accommodated into the *HTAB* micelles depends to the utmost extent on how well the chlorhexidine units and surfactant micelles fit together, considering their geometrical size and how favorable the mutual interactions between the components of the mixed micelles can be. Taking into account the first aspect, the length of the extended *CH^++^* conformation (considering the shape of the linear 1,6-hexamethylene linker), expressed as the Cl–Cl distance (ca. 2.4–2.9 nm, see Table S3, structures (1)–(3)), and the overall diameter of spherical *HTAB* micelles [128,129,130] (from ca. 5 to 6.6 nm within a temperature range of 25–40 °C) do not match at all. However, from this structural standpoint, *CH^++^* may adopt an overall V-shaped conformation (folded state, see Table S3, conformations (5)–(10)) able to be inserted into the *HTAB* micelles corona, as schemed simplistically in Figure 2. This possible mechanism was inspired by the interesting data published elsewhere [131,132]. Thus, these studies, based on neutron scattering measurements and molecular dynamics (carried out on the mixed systems of *CHDG*–phosphatidyl choline (*PC*) bilayers, with *CHDG* deuterium-labelled or unlabeled at the hexamethylene moiety), evinced convincingly both the place where *CHDG* entities are favorably located (near the headgroups region of the lipid bilayers) and the shape of *CHDG* (bent in half in a wedge-like fashion; Table S3, folded conformations). Moreover, the molecular dynamics simulations performed on *CH^++^* in water led to a broad distribution of Cl–Cl (belonging to the two chlorophenyl moieties of *CH^++^*) distance, while the same procedure performed in the *PC* bilayer exhibited a sharp distribution, with the most likely conformation having a Cl–Cl distance of ca. 1.5 nm [131]. These results support our assumption regarding the V-shaped conformations of *CH^++^* inserted into *HTAB* micelles. In this respect, the folded conformations (5)–(8) (Table S3) seem to best match the requirement of 1.5 nm for the Cl–Cl distance.

**Figure 7:**
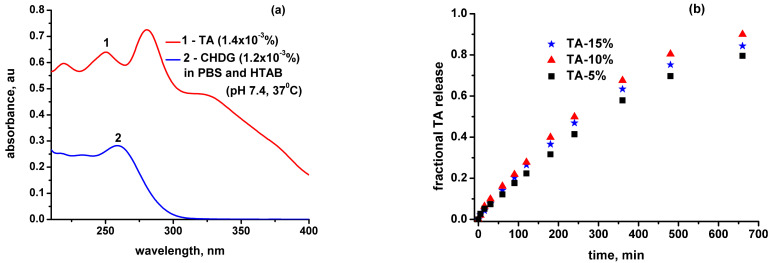
(**a**) UV–VIS absorption spectra for *TA* and *CHDG* in *PBS*–*HTAB* solution (pH 7.4, 37 °C) and (**b**) release of *TA* from *TA*–collagen matrices into the same solution.

The next step was to properly adjust a UV–VIS method for quantifying *TA/CHDG* released at a desired time with a satisfactory accuracy. In this way, analyzing UV–VIS absorption spectra of numerous TA/CHDG mixtures (with known compositions, see some in Figure S8), and based on the individual UV–VIS spectra of the releasing components in HTAB–sodium phosphate buffer (Figure 7a), it was found that the most reliable approach to estimate TA/CHDG amount released into the release environment is monitoring the values of absorbances at ca. 325 nm for TA and 260 nm for CHDG, respectively. Then, a subsequent experimental algorithm was obeyed to obtain TA/HTAB concentrations in release medium with good enough reliability and accuracy (for more details, see the text on page 13 with Figure S9 in Supplementary Materials). Briefly, this consisted of several main items:

- Construction of a data matrix containing on the upper line and left column the concentration values of TA and CH, covering the possible compositions compatible with the real experimental conditions (Table S2). Every single site of the bidimensional matrix represents a specific, discrete composition expressed as the concentration ratio of TA/CHDG in the releasing solution. Any composition obtained so, taken individually, should follow the Beer–Lambert law by diluting it correspondingly using the same releasing solvent (Figure S9);
- From the UV–VIS spectrum of a certain composition (extracted from the release medium at a desired time), the ratio of absorbances belonging to the two components (*A_325 nm_/A_260 nm_*), corrected by the corresponding extinction coefficients (found to be constant for a broad variety of TA–CHDG compositions), was compared to the values from the data matrix (Table S4). The closest value found in the table defines the relative composition most likely to be in the real system (extracted from the release medium);
- A series of 5–6 solutions of known concentrations (keeping the same ratio of *TA/CHDG*) were used to generate a calibration curve (a straight line, according to the Beer–Lambert law) on which the specific values of *A_325nm_* and *A_260nm_* allowed for finding the *TA* and *CHDG* concentrations in the real sample.

With the aid of this set of experimental rules, the time dependences of simultaneous *TA* and *CHDG* release have been obtained.

At the same time, the analysis of the overall kinetics of *TA*/*CHDG* release was based on some preliminary considerations allowing for the two particular cases of diffusional transport involved in the course of the releasing process. Therefore, the Fick’s second law of three-dimensional diffusion considering a constant diffusion coefficient (*D*) can be written as [133]:

(6) $$\frac{\partial c}{\partial t}=D\cdot \left(\frac{\partial^{2}c}{\partial x^{2}}+\frac{\partial^{2}c}{\partial y^{2}}+\frac{\partial^{2}c}{\partial z^{2}}\right)$$

where *c* is the concentration of a diffusing substance. In cylindrical coordinates, Equation (6) can be rewritten in accordance with the corresponding Laplacian operator form:

(7) $$\frac{\partial c}{\partial t}=D\cdot \left[\frac{1}{r}\cdot \frac{\partial }{\partial r}\left(r\cdot \frac{\partial c}{\partial r}\right)+\frac{1}{r^{2}}\cdot \frac{\partial^{2}c}{\partial \theta^{2}}+\frac{\partial^{2}c}{\partial z^{2}}\right]$$

where, generally, *r*∈[0,∞), *θ*∈[0,2π), and *z*∈(−∞,∞). Supposing a diffusion phenomenon occurring within a cylindrical volume (through the lateral area of the cylinder), it is useful to take into account the simplest case of radial diffusion within a plane located at a constant *z* coordinate and irrespective of the angular coordinate (*θ*). At given constant values for the quantities *z* and *θ*, Equation (7) defines so-called one-dimensional radial diffusion into a cylinder [133,134]:

(8) $$\frac{\partial c}{\partial t}=D\cdot \frac{1}{r}\cdot \frac{\partial }{\partial r}\left(r\cdot \frac{\partial c}{\partial r}\right)=D\cdot \left(\frac{1}{r}\cdot \frac{\partial c}{\partial r}+\frac{\partial^{2}c}{\partial r^{2}}\right)$$

For this kind of penetrant diffusion in a cylinder of radius *a*, the initial and boundary conditions, respectively, will be:

(9) $$c=c_{1},\;0<r<a,\;t=0$$

(10) $$c=c_{0},\;r=a,\;t>0$$

where *c_1_* is the constant diffusant concentration initially existing within the cylindrical volume (generally *c_1_* = 0) and *c_0_* is a constant concentration of diffusant located outside the cylinder.

Allowing for water (or solvent) uptake into a cylindrical sample, the concentrations defined above are referred to as the water/solvent (diffusant) concentration. A useful solution of Equation (8) under conditions (9) and (10) at “small times” has been demonstrated to be [134,135]:

(11) $$\frac{M_{t}}{M_{\infty }}=\frac{4}{\sqrt{\pi }}\cdot {\left(\frac{D\cdot t}{a^{2}}\right)}^{\frac{1}{2}}-\frac{D\cdot t}{a^{2}}-\frac{1}{3\sqrt{\pi }}\cdot {\left(\frac{D\cdot t}{a^{2}}\right)}^{\frac{3}{2}}+\ldots$$

where *M_t_* denotes the amount of water/solvent that has been retained by the cylinder in time, *t*, and *M_∞_* is the corresponding quantity at equilibrium (after infinite time). The term “small times” defines an approximation that is valid for the first 60% of water/solvent uptake (*M_t_/M_∞_* ≤ 0.6). Apart from the Fickian diffusion (Case I) described by Equation (11) for “small times”, Enscore et al. proposed a generalized model of the Case II transport in a polymeric sample based on its shape [136]:

(12) $$\frac{M_{t}}{M_{\infty }}=1-{\left(1-\frac{k_{0}}{c_{0}\cdot b}\cdot t\right)}^{N}$$

where *k*_0_ is the Case II relaxation constant of polymer. The quantity *b* is the half-thickness for planar samples or the radius of the cylinder/sphere (*a*) for cylindrical/spherical samples, respectively. Keeping the same order of sample geometry, the numerical values of *N* are 1 (for plane), 2 (for cylinder), or 3 (for sphere). Thus, the Case II water/solvent absorption by a cylindrical sample will be given by adapting Equation (12) accordingly:

(13) $$\frac{M_{t}}{M_{\infty }}=\frac{2k_{0}}{c_{0}\cdot a}\cdot t-{\left(\frac{k_{0}}{c_{0}\cdot a}\cdot t\right)}^{2}$$

By denoting *D_ap_*/*a*^2^ = *k*_0_/(*c*_0_·*a*), Equation (13) may be rewritten as:

(14) $$\frac{M_{t}}{M_{\infty }}=2\cdot \frac{D_{ap}}{a^{2}}\cdot t-{\left(\frac{D_{ap}}{a^{2}}\cdot t\right)}^{2}$$

where *D_ap_* is an apparent constant diffusion coefficient for the non-Fickian diffusion (Case II).

**Figure 8:**
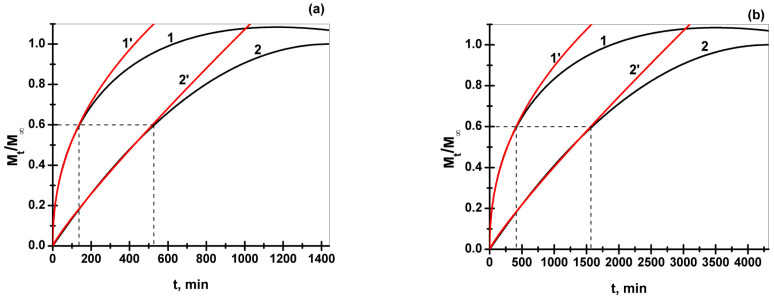
Time-dependent release for a Fickian (Equation (11), curves 1) and non-Fickian (Equation (14), curves 2) diffusion and the corresponding Peppas-type plots (Equation (15), curves 1′ and 2′) for the first 60% release of (**a**) *TA* and (**b**) *CHDG*.

The reverse phenomenon of the release of a solute contained into a cylindrical sample may be treated analogously, in accordance with Equations (11) and (14) by suitably redefining the significance of quantities *M_t_* and *M_∞_*: *M_t_*—the amount of solute released from the sample up to time, *t*, and *M_∞_*—the amount of solute desorbed into the releasing environment after infinite time. Depending on the type of solute loaded into the cylindrical sample (in our case, *TA* and *CHDG*, respectively), the times at which *M_t_* reaches its equilibrium values (when fractional release is considered to be practically equal to unity) differ significantly. Thus, a time period of 24 h (1440 min) to attain the equilibrium release for *TA* was experimentally observed, and 72 h (4320 min) for *CHDG*. As a result, the two types of solute release described by Equations (11) and (14) require two distinct values for the quantity *D_ap_/a^2^*: 1/1440 min^−1^ for *TA* and 1/4320 min^−1^ for *CHDG* release, respectively (Figure 8).

The real release behavior of a solute contained by a real polymer-based matrix does strictly obey neither Fickian nor non-Fickian diffusion (Case II) but follows an intermediate mechanism. Thus, Peppas et al. [111,134,137,138] proposed an empirical equation, so-called the power law model, as under:

(15) $$\frac{M_{t}}{M_{\infty }}=k\cdot t^{n}$$

where *k* is a kind of kinetic constant related to both the structural and geometrical features of the matrix and *n*-diffusional exponent that is an indicator of the transport/diffusion mechanism of the solute/drug through the swollen matrix. When *n* = 0.5, the release of solute is considered Fickian, as demonstrated for diffusion from a plane matrix, based on Fick’s laws [111,134]. Moreover, by accounting the same system of drug/solute release, the Case II transport may be carried out when *n* = 1. For the real matrices, the release of solute/drug is a result of the coupling of diffusion and relaxation phenomena, both of them leading to a non-Fickian (anomalous) intermediate behavior. While the specific values of *n* for a plane, very thin matrix are 0.5 and 1.0 for Fickian and non-Fickian (Case II) diffusion behavior, respectively, by changing the shape of the delivery system into a cylindrical one, these values become 0.45 and 0.89, respectively [134,139]. Any other values of *n* within the 0.45–0.89 range define the so-called anomalous diffusion. In our specific case, we performed the release experiments upon a cylinder-shaped matrix, with the upper and bottom face of the cylinder covered by a thin wax layer as a water-repellent barrier. Indeed, by fitting the first 60% of solute release shown in Figure 8 (curves 1 and 2) with Equation (15) (red curves 1′ and 2′), the abovementioned values of *n* were fully confirmed (both *n* and *k* values are collected in Table 4).

The release data for all the collagen-based matrices prepared and entirely fitted with only the Peppas-type Equation (15) are summarized in Tables S5–S7. However, for a preliminary assessment of *TA*/*CHDG* release, it was useful to establish the contribution of each type of diffusion mechanism to the overall solute release. Accordingly, any fractional release-time dependence (up to 0.6 fractional release) may be written as a weighted sum of the two types of diffusion mechanisms as follows:

(16) $$\frac{M_{t}}{M_{\infty }}=k\cdot t^{n}=a\cdot 0.06569\cdot t^{0.4497}+b\cdot 0.00234\cdot t^{0.88703}$$

for *TA* release and

(17) $$\frac{M_{t}}{M_{\infty }}=k\cdot t^{n}=a\cdot 0.04009\cdot t^{0.4497}+b\cdot 0.00088\cdot t^{0.88703}$$

for *CHDG* release.

Before discussing the release behavior from the collagen-based matrices, it is worth noting for the purpose of the present work that all the values of diffusional exponents fall within 0.45–0.89, which demonstrates an anomalous transport mechanism for both *TA* and *CHDG* during the releasing process.

As for *TA* release from the *TA*–collagen matrices, all four (a)–(d) factors abovementioned seem to have influenced the process. Referring back to the preparation of porous *TA*–collagen matrices from corresponding acidic hydrogels presented elsewhere [140] (see Figure 6), it became clear enough that the crosslinker role of *TA* was achieved mainly by hydrogen bonding between the polyphenolic *–OH* groups of *TA* and *–COOH*, *–OH*, and peptide groups belonging to collagen, on one hand, and by Yoshida-type H-bonding previously discussed. Consequently, at a low *TA* concentration (5%), the majority of *TA* may be bound onto collagen fibrils/molecules. As a result, a low release rate may occur (Figure 7b). Increasing the *TA* concentration to 10%, the amount of “nonbound” *TA* increased, becoming more available to be solubilized by the *HTAB* micelles of the release solution. That is why the relative amount of *TA* released also increased and the time dependence release for 10% *TA* is placed above that for 5% *TA*. The amount of *TA* released per unit area per unit time continued to intensify until reaching its maximum value imposed by the *TA* solubilization rate into *HTAB* micelles. Beyond this point, as in the case of the collagen matrix with 15% *TA*, the process practically became rate-controlled. Thus, the relative amount of *TA* released per unit area per unit time (relative flux) diminished, even though the release flux (not relative) remained constant. It is also worth mentioning that the non-Fickian mechanism of TA release is by far prevalent, with relative weights (based on the value of dimensionless coefficients *a* and *b* in Equation (16)) easily exceeding 90%, as exhibited in Table S8 (Supplementary Materials). This behavior is consistent with an anomalous Fickian transport much closer to the Case II mechanism as mirrored by the values of general diffusion exponent (*n*) ranging between 0.792 and 0.846 (Table S8). Instead, by fitting the entire experimental dataset of swelling (not only those corresponding to the first 60% of *TA* release as above) with the Peppas-type Equation (15), the values of diffusional exponent descended significantly (0.686–0.735, Table S5), which might suggest a lower contribution of relaxation phenomena of the collagen-based network to the releasing process.

The presence of *CHDG* drastically changed *TA* release from the *TA*–*CHDG*–collagen matrices. Thus, for the matrices with the minimum *CHDG* content (1.82%, see Figure S10a), the most significant factor seems to be (b) *CHDG* interaction with the collagenous substrate under release conditions. Indeed, collagen molecules of the swollen matrix (pH 7.4) became negatively charged. This is because the extraction technology of collagen extraction performed by our supplier involved an intermediate stage of sodium hydroxide treatment. As a result, some asparagine and glutamine residues of collagen might have undergone deamidations, leading to the corresponding aspartate and glutamate residues. These newly-formed amino acid residues could have been responsible for lowering the isoelectric point of collagen into an acidic domain (pH 4.6–6.0), as reported elsewhere [57]. Thus, electrostatically induced interactions between chlorhexidine dication and negatively charged collagen (at pH 7.4) significantly altered the collagenous substrate with respect to *TA*. The direct consequence was a low *TA* binding (and low degree of *TA* binding) onto collagen. In this way, based on the experimental data (Figure S10a), *TA* release did not depend to a large extent on its loading concentration inside the matrix, but mainly on the solubilization rate in *HTAB* micelles. Such a rather rate-controlled process is equivalent to an almost constant release flux of *TA* (irrespective of its concentration) and decrease in its relative release as *TA* content ascends from 5 to 15%. This behavior is also mirrored by the majority proportion of Case II diffusion (with a percentage of non-Fickian Case II behavior between ca. 63 and 76% as shown in Table S9). However, a relative decreasing contribution of the collagen network relaxation to the transport mechanism is sustained by the diffusion exponent values (0.595, 0.628, 0.673) closer to 0.45 than to 0.89 when it comes to a fractional *TA* release up to 60% (Table S7). This course is supported by the values of *n* (0.562, 0.628, 0.630) being a little bit smaller when 100% of releasing data were considered.

Similarly, the crosslinking effect induced by *CH* onto the collagen network as a result of attractive electrostatic interactions *CH^++^*-negatively charged collagen led to the same relatively low degree of *TA* binding on collagen for *TA–CHDG*(4.55%)–collagen matrices, as just described for *TA–CHDG*(1.82%)–collagen sponges. At the same time, the rate-controlled process of *TA* release imposed by the same rate of *TA* solubilization by *HTAB* micelles gave a roughly similar descendant order of time dependent *TA* releases when *TA* load increased from 5 to 15% (Figure S10b). The kinetics data obeyed the tendency noticed in the case of previous *TA–CHDG*–collagen sponges loaded with 1.82% *TA*, which practically reveals the prevailing proportion of the non-Fickian Case II diffusion (Table S9) corresponding to a general anomalous transport mechanism (Table S6) for *TA* release in the aqueous release medium.

The possibility of mixed *HTAB*-based micelles to enhance continuously their ability of *TA* solubilization with increasing *CH* content, coupled with the formation of a *TA–CH* complex, could be a realistic one when inspecting TA release from porous *TA*–*CHDG* (9.09%)–collagen matrices. Actually, the structural evolution of the mixed *TA–CH–HTAB* micelles should be strongly related to the amount of *CH* embedded, which, in turn, may generate thermodynamic instability of micellar assemblage due to the repulsive interactions between the cationic heads of the surfactant and *CH^++^* dications. Accordingly, to improve thermodynamic stability, such micellar structures require insertion of additional *TA* entities as relatively non-charged or partially charged with negative charges spacers (see the scheme of process in Figure 2). Indeed, a general value of protonated constant (acidity exponent, *pK_a_*) estimated by Ghigo et al. [141] for *TA* is of 7.5, which is equivalent to say that about half the *TA* species are statistically in protonated form (non-charged) and the rest of them are in the deprotonated state (negatively charged), favoring mixed micelles formation. At the same time, the similarity in ionic size of *Br^−^* (0.196 nm in radius) and hexadecyltrimethylammonium cation (*HTA^+^*, considered to possess a radius close to that of tetramethylammonium cation, ca. 0.2 nm) [142], as parts of every single *HTAB* species, partially lead to development of undissociated inner sphere ion pairs *HTA^+^/Br^−^* in an aqueous solution according to the Collins’ theory of matching water affinity with respect to a given cation–anion pair [143,144]. Such a behavior is partly supported by a value of 0.42 conductometrically determined in pure water for the degree of counterions (bromides) binding in *HTAB* micelles [145]. This phenomenon determines not only an increase in micelle size, but also an enhanced capacity of *TA* solubilization by the newly mixed micelles when *CH* content becomes greater.

Secondly and simultaneously, a possible nonstoichiometric *TA–CHDG* complex formation (due to reversible electrostatic interactions between *CH^++^* and negatively charged *TA* at pH 7.4, Yoshida H-bonding, and π-π stacking between *TA* and *CHDG*) might have played a significant role in altering *TA* release from three-component matrices with a maximum content of *CHDG* (9.09%):

(18) $$\mathrm{nTA}+\mathrm{mCHDG}⇄{\left(\mathrm{AT}\right)}_{n}{\left(\mathrm{CHDG}\right)}_{m}$$

where *n* and *m* are unknown compositional indices (probably *m ≥ n*). Thus, we hypothesized that at a large *CHDG* content, a certain amount of *TA* could have been strongly “immobilized” in the complex *TA–CHDG* formed. Typically, at low level of *TA*, most of the “immobilized” *TA* was more difficult to solubilize in the mixed *HTAB*-based micelles, which could explain the lowest fractional release (Figure S10c) over the entire time period of data acquisition (660 min). On the other hand, the increasing capacity of the mixed *HTAB* micelles (able to incorporate much more *CH*) could have led to an enhanced solubilization of *TA* when its percentage was 10 or 15% in a way somehow proportional to the available (less “immobilized”) *TA* content. Such a behavior could explain the high similarity of *TA* release traces when its concentration increased and also the greater fractional release when compared to the system *TA*(5%)–*CHDG*(9.09%)–collagen.

From a kinetic point of view, the prevailing Case II diffusion (involving the relaxation contribution of the collagen network) over the Fickian transport mechanism is in line with the similar peculiarity noticed in the other cases of *TA* release from *TA–CHDG*–collagen matrices considered up to 60% of fractional release (Table S9). This aspect is asserted by the corresponding anomalous mechanism of *TA* diffusion outside the matrix into the releasing environment, when all the dataset of *TA* release fitted with the Peppas-like Equation (15) led to a diffusional index of about 0.6 (Table S6).

The most important features concerning *CHDG* release from *TA*/*CHDG*–collagen matrices are reflected in (a) maintaining the release dependences very close to each other (practically superimposed) at a certain *CHDG* concentration and different *TA* contents (see Figure S10a–c) and (b) a well-defined order of *CHDG* release dependences from the matrices where *TA* concentration was fixed and *CHDG* amount was changed. The first aspect confirms the predominance of chlorhexidine dication–collagen interaction over the *TA*–collagen one under releasing conditions (pH 7.4, 37 °C). Therefore, the rate of relative *CHDG* release is mainly determined by the strength of *CHDG* binding (electrostatic in nature) onto the collagenous swollen matrix. On the other hand, the solubilization capacity of the mixed micelles seems not to influence *CHDG* release. This is supported by the decreasing tendency in *CHDG* release when the *CHDG* concentration varies from 1.82% *CHDG* to 9.09% at the same *TA* content (Figure S6a–c). The behavior experimentally observed is indicative of the existence of a rate-controlled transport process, irrespective of *CHDG* content. Consequently, the absolute release flux of *CHDG* into the release medium at a certain moment (taken the same for all the systems) is practically the same for all *TA*–*CHDG*–collagen matrices, which means that the relative amount of *CHDG* released at that time decreases as *CHDG* concentration rises. In all these cases of chlorhexidine release (simultaneously with tannic acid), the corresponding three-component collagen-based matrices have shown an important contribution derived from the relaxation of the collagen network during the anomalous diffusion, as can be seen in Table S10 (contribution of the Case II diffusion was found to be of ca. 64–78%, with a value of diffusional index of about 0.6 by considering the first 60% of *CHDG* release) and Table S7 (where the general quantity *n*, by taking into account the entire *CHDG* releasing process, has a similar value of ca. 0.6).

## 4. Conclusions

The in vitro simultaneous release of *TA* and *CHDG* from three-component *TA–CHDG*–atelocollagen type I porous matrices into a solution of *PBS* containing cationic micellar carriers of *HTAB* (pH 7.4, 37 °C):

- Was monitored spectrophotometrically (UV–VIS) according to an original algorithm designed and successfully applied;
- Was kinetically studied and, to the best of our knowledge, an adapted kinetic approach developed in this study led, for the first time, to general results of anomalous diffusion able to be rationally split into the two contribution parts related to the pure Fickian and non-Fickian Case II regime of diffusion/release, respectively;
- Was strongly influenced, and eventually able to be controlled, by both the density of non-covalent crosslinkages of the collagen network produced by *TA* and *CHDG* and the dynamic capacity of *HTAB* micelles contained in the release environment to solubilize *TA* and *CHDG*.

## Figures and Tables

**Table 1:** Characteristic infrared bands for the collagen-based matrices with *TA* (5, 10, 15%) and *TA* (5, 10, 15%)–*CHDG* (9.09%).

Designation	Collagen	Collagen *TA*–5%	Collagen *TA*–10%	Collagen *TA*–15%	Collagen *TA*–5% *CHDG*–9.09%	Collagen *TA*–10% *CHDG*–9.09%	Collagen *TA*–15% *CHDG*–9.09%	Assignment
-	3455	3485	3495	3500	3485	3490	3500	(hydrogen bond) hydrogen bonded water
Amide A	3301	3292	3297	3291	3306	3304	3297	NH stretching/C=N (guanidine) symmetrical stretching
Amide B	3077	3078	3081	3079	3081	3081	3087	NH stretching
-	2933	2937	2929	2940	2933	2933	2938	CH_2_ asymmetrical stretching
-	2873	2876	2876	2881	2876	2876	2876	CH_2_ symmetrical stretching CH_3_ asymmetrical stretching
Amide I	1630	1630	1630	1631	1631	1632	1631	C=O stretching
Amide II	1545	1542	1539	1539	1539	1539	1536	CN stretching, NH bending
-	1450	1447	1447	1447	1449	1448	1447	CH_2_ bending
-	1339	1336	1333	1333	1337	1336	1335	in plain OH (phenol) bending
Amide III	1236	1234	1232	1232	1237	1235	1234	CN stretching, NH bending, CH_2_ wagging, CN stretching (guanidine)
-	1204	1203	1201	1200	1204	1202	1202	C-O stretching (C-OH in phenol)
-	1080	1080	1080	1079	1081	1080	1080	C-O stretching (carbohydrate moiety)/C-O-C asymmetrical stretching
-	1033	1033	1033	1033	1033	1033	1033	C-O stretching (carbohydrate moiety)/C-O-C symmetrical stretching
ν_amideI_-ν_amideII_	85	88	91	92	92	93	95	
A_amide III_/A_1450_	0.9	0.9	0.9	0.9	0.9	0.9	0.9	

**Table 2:** Estimation of the degree of crosslinking (*DC*) of some collagen matrices based on swelling (in *PBS-HTAB*, pH 7.4 at 37 °C) data at equilibrium according to Equation (2)—case 1: negligible amounts of *TA* and *CHDG* were released at equilibrium.

System	ρ_matrix_, g/cm^3^	$Q_{w,\mathrm{matrix}}^{\mathrm{eq}}$, g/g	$Q_{v,\mathrm{matrix}}^{\mathrm{eq}}{/Q}_{v,\mathrm{coll}}^{\mathrm{eq}}$, *v/v*	ν_2eq_	c_p_ × 10^8^, mol/cm^3^	Δc × 10^7^, mol/cm^3^	DC × 10^5^, mol/cm^3^
Collagen	1.195	6.80	7.34/7.34	0.136	953	2.5	58
Collagen–TA (5%)	1.245	5.66	6.31/6.46	0.155	1087	3.3	86
Collagen–TA (10%)	1.290	4.30	4.89/5.11	0.196	1374	5.2	184
Collagen–TA (15%)	1.316	4.11	4.74/5.06	0.198	1388	5.3	191
Collagen–TA (5%)-CHDG (9.09%)	1.248	4.06	4.49/4.92	0.203	1423	5.6	208
Collagen–TA (10%)-CHDG (9.09%)	1.285	4.02	4.55/5.09	0.196	1374	5.2	184
Collagen–TA (15%)-CHDG (9.09%)	1.319	4.16	4.81/5.50	0.182	1276	4.5	144

**Table 3:** Estimation of the degree of crosslinking (*DC*) of some collagen matrices based on swelling (in *PBS–HTAB*, pH 7.4 at 37 °C) data at equilibrium according to Equation (2)—case 2: both *TA* and *CHDG* were almost majority released at equilibrium.

System	ρ_matrix_, g/cm^3^	$Q_{w,\mathrm{coll}}^{\mathrm{eq}}$, g/g	$Q_{v,\mathrm{coll}}^{\mathrm{eq}}$, *v/v*	ν_2eq_	c_p_ × 10^8^, mol/cm^3^	Δc × 10^7^, mol/cm^3^	DC × 10^5^, mol/cm^3^
Collagen	1.195	6.80	7.34	0.136	953	2.5	58
Collagen–TA (5%)	1.245	5.66	6.09	0.164	1150	3.7	103
Collagen–TA (10%)	1.290	4.30	4.61	0.217	1521	6.4	261
Collagen–TA (15%)	1.316	4.11	4.40	0.227	1591	7.0	306
Collagen–TA (5%)-CHDG (9.09%)	1.248	4.06	4.35	0.230	1612	7.2	321
Collagen–TA (10%)-CHDG (9.09%)	1.285	4.02	4.30	0.233	1633	7.4	336
Collagen–TA (15%)-CHDG (9.09%)	1.319	4.16	4.45	0.225	1577	6.9	297

**Table 4:** Calculated values of kinetic constants (*k*), diffusional exponents (*n*), and coefficients of determination (*R^2^*) for Fickian and non-Fickian Case II release (up to 0.6) of *CHDG* and *TA* from a cylinder-shaped matrix.

Solute	Case I			Case II		
	k × 10^2^, min^−n^	n	R^2^	k × 10^3^, min^−n^	n	R^2^
*TA*	6.569 ± 0.087	0.4497 ± 0.0030	0.99765	2.34 ± 0.02	0.88703 ± 0.00167	0.99938
*CHDG*	4.009 ± 0.066	0.4497 ± 0.0030	0.99765	0.88 ± 0.01	0.88703 ± 0.00167	0.99938

## Data Availability

Not applicable.

## Supplementary Materials

The following supporting information can be downloaded at: https://www.mdpi.com/article/10.3390/pharmaceutics15010076/s1. 10 tables ((1) Table S1: Characteristic infrared bands for some collagen-based matrices; (2) Table S2: The amino acid residues and their features mainly involved in generating the fixed electrical charges borne by a single triple helix of collagen type I extracted from calf skin; (3) Table S3: Planar and spatial structures of some extended and folded conformers/tautomers of chlorhexidine dication, highlighting the distances between chlorine atoms; (4) Table S4: The values matrix for the TA/CHDG compositions used to guide the experimental measurements. The values inside the nonbolded cells are the ratios of TA to CHDG concentrations; (5) Table S5: The values of the kinetic constants (k), diffusional exponents (n), coefficients of determination (R^2^) and fractional releases after 11 h (c_11_) for the entirely (up to 100%) determined TA release from the TA-collagen matrices; (6) Table S6: The values of the kinetic constants (k), diffusional exponents (n), coefficients of determination (R^2^) and fractional releases after 11 h (c_11_) for the entirely (up to 100%) determined TA release from the TA-CHDG-collagen matrices; (7) Table S7: The values of the kinetic constants (k), diffusional exponents (n), coefficients of determination (R^2^) and fractional releases after 11 h (c_11_) for the entirely (up to 100%) determined CHDG release from the TA-CHDG-collagen matrices; (8) Table S8: The values of the kinetic constants (k), diffusional exponents (n), coefficients of determination (R^2^) and weights of (a) Fickian and (b) non-Fickian-Case II diffusion for the TA release (up to 0.6) from the TA-collagen matrices; (9) Table S9: The values of the kinetic constants (k), diffusional exponents (n), coefficients of determination (R^2^) and weights of (a) Fickian and (b) non-Fickian-Case II diffusion for the TA release (up to 0.6) from the TA-CHDG-collagen matrices; (10) Table S10: The values of the kinetic constants (k), diffusional exponents (n), coefficients of determination (R^2^) and weights of (a) Fickian and (b) non-Fickian-Case II diffusion for the CHDG release (up to 0.6) from the TA-CHDG-collagen matrices), 10 figures ((11) Figure S1: FT-IR spectra (extended and fingerprint region) for the collagen-based matrices with the specified compositions (CHDG-free and 9.09% CHDG); (12) Figure S2: FT-IR spectra (extended and fingerprint region) for the collagen-based matrices with the specified compositions (4.55% CHDG); (13) Figure S3: FT-IR spectra (extended and fingerprint region) for the collagen-based matrices with the specified compositions (1.82% CHDG); (14) Figure S4: SEM micrographs (taken from sections parallel to air-sided face) for (a) collagen matrix and collagen-based matrices with (b) TA(10%) and with (c) TA(10%)-CHDG(9.09%); (15) Figure S5: Estimated temperature evolution of buffer density (upper graph) obeying the same tendency as for water density (bottom plot) (buffer density of 1.098 g/cm^3^ at 20 °C was pycnometrically determined) (see www.vip-ltd.co.uk for water density values; accessed on November 19, 2022); (16) Figure S6: CHDG release from the collagen-based matrices with (a) 5% TA, (b) 10% TA and (c) 15% TA into PBS-HTAB solution (pH 7.4) at 37 °C; (17) Figure S7: Concentration dependence of I_1_/I_3_ of Py in HTAB solution at 37 °C (5 × 10^−7^ M Py in 10 mM buffer phosphate); (18) Figure S8: Changes in UV-VIS spectra of TA in different aqueous environments (a–d), the corresponding spectra of TA and CHDG (taken separately) in PBS and HTAB (e) and the UV-VIS spectra for three TA-CHDG mixtures in the same release solution (PBS+HTAB) at the specified compositions (f); (19) Figure S9: (a) Obeying Lambert-Beer’s law starting from (b) an initial solution of minimum concentration of 2.03 × 10^−4^% TA and 2.9 × 10^−4^% CHDG and a final solution of maximum concentration of 1.20 × 10^−3^% TA and 1.71 × 10^−3^% CHDG in PBS and HTAB with keeping the concentration ratio constant (TA/CHDG – 0.7; the arrow indicates how components concentrations increase); (20) Figure S10: TA (filled symbols) and CHDG (open symbols) release from collagen-based matrices into PBS-HTAB solution (pH 7.4) at 37 °C) and 4 text sections ((17) Determination of cmc of HTAB in 10 mM sodium buffer phosphate at 37 °C by using I_1_/I_3_ ratio of pyrene; (19) Experimental algorithm on determination of TA/CHDG concentrations in release medium; (21) Ionic strength and temperature effects on practical/apparent acid exponent *pK_ap_*; (22) Relationships used for defining some quantities at equilibrium swelling required in the evaluation of crosslinking degree associated with the studied collagen-based matrices)).

## References

[1] Sezer A.D., Cevher E., Pignatello R. (2011). Biopolymers as Wound Healing Materials: Challenges and New Strategies. Biomaterials Applications for Nanomedicine.

[2] Seaman S. (2002). Dressing Selection in Chronic Wound Management. J. Am. Podiatr. Med. Assoc..

[3] Lee C.H., Singla A., Lee Y. (2001). Biomedical applications of collagen. Int. J. Pharm..

[4] Friess W. (1998). Collagen—Biomaterial for drug delivery. Eur. J. Pharm. Biopharm..

[5] Yannas I.V. (1990). Biologically Active Analogues of the Extracellular Matrix: Artificial Skin and Nerves. Angew. Chem. Int. Ed..

[6] Pachence J.M., Berg R.A., Silver F.H. (1987). Collagen: Its place in the medical device industry. Med. Device Diagn. Ind..

[7] Hayward P.G., Morrison W.A. (1996). Current concepts in wound dressings. Aust. Prescr..

[8] Sripriya R., Kumar M.S., Sehgal P.K. (2004). Improved collagen bilayer dressing for the controlled release of drugs. J. Biomed. Mater. Res..

[9] Stemberger A., Grimm H., Bader F., Rahn H.D., Ascherl R. (1997). Local treatment of bone and soft tissue infections with the collagen-gentamicin sponge. Eur. J. Surg. Suppl..

[10] Rutten H.J.T., Nijhuis P.H.A. (1997). Prevention of wound infection in elective colorectal surgery by local application of gentamicin-containing collagen sponge. Eur. J. Surg. Suppl..

[11] Chokotho L., Van Hasselt E. (2005). The use of tannins in the local treatment of burn wounds—A pilot study. Malawi Med. J..

[12] de Maquinarias V., Monografias M. (2001). The use of tannic acid in the local treatment of burn wounds: Intriguing old and new perspectives. Wounds.

[13] Davidson E.C. (1925). Tannic acid in the treatment of burns. Surg. Gynecol. Obstet..

[14] Haslam E. (1989). Plant Polyphenols: Vegetable Tannins Revisited.

[15] Meek K.M., Weiss J.B. (1979). Differential fixation of poly(L-arginine) and poly(L-lysine) by tannic acid and its application to the fixation of collagen in electron microscopy. Biochim. Biophys. Acta.

[16] Futaesaku Y., Mizuhira V., Nakamura H. (1972). The new fixation method using tannic acid for electron microscope and some observation of biological specimens. Proc. Int. Congr. Histochem. Cytochem. Kyoto.

[17] Madhan B., Dhathathreyan A., Subramanian V., Ramasami T. (2003). Investigations on geometrical features in induced ordering of collagen by small molecules. J. Chem. Sci..

[18] Velmurugan P., Singam E.R.A., Jonnalagadda R.R., Subramanian V. (2014). Investigation on interaction of tannic acid with type I collagen and its effect on thermal, enzymatic, and conformational stability for tissue engineering applications. Biopolymers.

[19] Tang H.R., Covington A.D., Hancock R.A. (2003). Structure-activity relationships in the hydrophobic interactions of polyphenols with cellulose and collagen. Biopolymers.

[20] Oh H.I., Hoff J.E., Armstrong G.S., Haff L.A. (1980). Hydrophobic interaction in tannin-protein complexes. J. Agric. Food Chem..

[21] Charulatha V., Rajaram A. (2003). Influence of different crosslinking treatments on the physical properties of collagen membranes. Biomaterials.

[22] Demeter M., Meltzer V., Calina I., Scarisoreanu A., Micutz M., Albu Kaya M.G. (2020). Highly elastic superabsorbent collagen/PVP/PAA/PEO hydrogels crosslinked via e-beam radiation. Rad. Phys. Chem..

[23] Demeter M., Călina I., Scărișoreanu A., Micutz M., Kaya M.A. (2022). Correlations on the Structure and Properties of Collagen Hydrogels Produced by E-Beam Crosslinking. Materials.

[24] Demeter M., Călina I., Scărișoreanu A., Micutz M. (2021). E-Beam Cross-Linking of Complex Hydrogels Formulation: The Influence of Poly(Ethylene Oxide) Concentration on the Hydrogel Properties. Gels.

[25] Micutz M., Lungu R.M., Circu V., Ilis M., Staicu T. (2020). Hydrogels Obtained via *γ*-Irradiation Based on Poly(Acrylic Acid) and Its Copolymers with 2-Hydroxyethyl Methacrylate. Appl. Sci..

[26] Călina I., Demeter M., Scărișoreanu A., Micutz M. (2021). Development of Novel Superabsorbent Hybrid Hydrogels by E-Beam Crosslinking. Gels.

[27] Chen C., Yang H., Yang X., Ma Q. (2022). Tannic acid: A crosslinker leading to versatile functional polymeric networks: A review. RSC Adv..

[28] Baldwin A., Uy L., Booth B.W. (2021). Characterization of collagen type I/tannic acid beads as a cell scaffold. J. Bioact. Compat. Polym..

[29] Michalska-Sionkowska M., Warzynska O., Kaczmarek-Szczepanska B., Łukowicz K., Osyczka A.M., Walczak M. (2021). Characterization of collagen/beta glucan hydrogels crosslinked with tannic acid. Polymers.

[30] Baldwin A., Uy L., Frank-Kamenetskii A., Strizzi L., Booth B.W. (2020). The in vivo biocompatibility of novel tannic acid-collagen type I injectable bead scaffold material for breast reconstruction post-lumpectomy. J. Biomater. Appl..

[31] Reed E.B., Ard S., La J., Park C.Y., Culligan L., Fredberg J.J., Smolyaninova L.V., Orlov S.N., Chen B., Guzy R. (2019). Anti-fibrotic effects of tannic acid through regulation of a sustained TGF-beta receptor signaling. Respir. Res..

[32] Cass C.A.P., Burg K.J.L. (2012). Tannic acid cross-linked collagen scaffolds and their anti-cancer potential in a tissue engineered breast implant. J. Biomater. Sci. Polym. Ed..

[33] Wang R., Yu R., Wang J., Xiang J., Chen C., Liu G., Liao X. (2022). Hierarchical collagen fibers complexed with tannic acid and Fe3+ as a heterogeneous catalyst for enhancing sulfate radical-based advanced oxidation process. Environ. Sci. Pollut. Res..

[34] Wu J., Liao W., Zhang J., Chen W. (2019). Thermal behavior of collagen crosslinked with tannic acid under microwave heating. J. Therm. Anal. Calorim..

[35] Ngobili T.A., Shah H., Park J.P., Kwist K.W., Inskeep B., Burg K.J.L., Booth B.W. (2015). Remodeling of tannic acid crosslinked collagen type I induces apoptosis in ER^+^ breast cancer cells. Anticancer Res..

[36] Pilloni A., Ceccarelli S., Bosco D., Gerini G., Marchese C., Marini L., Rojas M.A. (2021). Effect of chlorhexidine digluconate in early wound healing of human gingival tissues. A histological, immunohistochemical and biomolecular analysis. Antibiotics.

[37] Gränicher K.A., Karygianni L., Attin T., Thurnheer T. (2021). Low concentrations of chlorhexidine inhibit the formation and structural integrity of enzyme-treated multispecies oral biofilms. Front. Microbiol..

[38] Pratt R., Pellowe C., Wilson J., Loveday H., Harper P., Jones S., McDougall C., Wilcox M. (2007). epic2: National Evidence-Based Guidelines for Preventing Healthcare-Associated Infections in NHS Hospitals in England. J. Hosp. Infect..

[39] Filoche S., Soma K., Sissons C.H. (2005). Antimicrobial effects of essential oils in combination with chlorhexidine digluconate. Oral Microbiol. Immunol..

[40] O’Grady N.P., Alexander M., Dellinger E.P., Gerberding J.L., Heard S.O., Maki D.G., Masur H., McCormick R.D., Mermel L.A., Pearson M.L. (2002). Guidelines for the prevention of intravascular catheter-related infections. Centers for disease control and prevention. MMWR Recomm. Rep..

[41] McDonnell G., Russell A.D. (1999). Antiseptics and disinfectants: Activity, action, and resistance. Clin. Microbiol. Rev..

[42] Jones C.G. (1997). Chlorhexidine: Is it still the gold standard?. Periodontology.

[43] Wakshlak R.B.-K., Pedahzur R., Menagen B., Avnir D. (2016). An antibacterial copper composite more bioactive than metallic silver. J. Mater. Chem. B.

[44] Nagarajan S., Soussan L.S., Bechelany M.B., Teyssier C.T., Cavaillès V.C., Pochat-Bohatier C., Miele P., Kalkura N., Janot J.-M., Balme S. (2016). Novel biocompatible electrospun gelatin fibers mat with antibiotic drug delivery properties. J. Mater. Chem. B.

[45] Al-Obaidy S.S.M., Greenway G.M., Paunov V.N. (2021). Enhanced antimicrobial action of chlorhexidine loaded in shellac nanoparticles with cationic surface functionality. Pharmaceutics.

[46] Al-Awady M.J., Weldrick P.J., Hardman M.J., Greenway G.M., Paunov V.N. (2018). Amplified antimicrobial action of chlorhexidine encapsulated in PDAC-functionalized acrylate copolymer nanogel carriers. Mater. Chem. Front..

[47] Cai X., Han B., Liu Y., Tian F., Liang F., Wang X. (2017). Chlorhexidine-loaded amorphous calcium phosphate nanoparticles for inhibiting degradation and inducing mineralization of type I collagen. ACS Appl. Mater. Interfaces.

[48] Rudolf J.-L., Moser C., Sculean A., Eick S. (2019). In-vitro antibiofilm activity of chlorhexidine digluconate on polylactide-based and collagen-based membranes. Oral Health.

[49] Coquet L., Obry A., Borghol N., Hardouin J., Mora L., Othmane A., Jouenne T. (2017). Impact of chlorhexidine digluconate and temperature on curli production in Escherichia coli—Consequence on its adhesion ability. AIMS Microbiol..

[50] Nomura R., Inaba H., Matayoshi S., Yoshida S., Matsumi Y., Matsumoto-Nakano M., Nakano K. (2020). Inhibitory effect of a mouth rinse formulated with chlorhexidine gluconate, ethanol, and green tea extract against major oral bacterial species. J. Oral Sci..

[51] Wakshlak R.B.-K., Pedahzur R., Avnir D. (2019). Antibacterial Activity of Chlorhexidine-Killed Bacteria: The Zombie Cell Effect. ACS Omega.

[52] Karpanen T.J., Worthington T., Hendry E.R., Conway B.R., Lambert P.A. (2008). Antimicrobial efficacy of chlorhexidine digluconate alone and in combination with eucalyptus oil, tea tree oil and thymol against planktonic and biofilm cultures of Staphylococcus epidermidis. J. Antimicrob. Chemother..

[53] Suresh B., Sriram S., Dhanaraj S., Elango K., Chinnaswamy K. (1997). Anticandidal activity of Santolina chamaecyparissus volatile oil. J. Ethnopharmacol..

[54] Boruziniat A., Babazadeh M., Gifani M., Nasirzadeh M. (2017). Effect of tannic acid application on durability of bond of etch and rinse adhesive resins. J. Dent. Mater. Tech..

[55] Netmeds Chlorhexidine 0.2% + Tannic Acid 3% + Zinc Chloride 1%. https://www.netmeds.com/generics/chlorhexidine-0-2-tannic-acid-3-zinc-chloride-1-.

[56] Şulea D., Ghica M.V., Micutz M., Albu M.G., Brăzdaru L., Staicu T., Leca M., Popa L. (2010). Characterization and in vitro release of chlorhexidine digluconate comprised in type I collagen hydrogels. Rev. Roum. Chim..

[57] Şulea D., Micutz M., Albu M.G., Staicu T., Leca M. (2011). Collagen-thuja tincture biomaterials for wound treatment. 2. Hydrogels and porous matrices. Rev. Roum. Chim..

[58] Micutz M., Staicu T., Leca M., Ghica C. (2009). Adsorption complexes of collagenous polypeptide-ionic surfactant in aqueous medium. 1. The formation of micellar structure of ionic surfactant adsorbed onto collagenous polypeptide chain. Rev. Roum. Chim..

[59] Meng Z., Zheng X., Tang K., Liu J., Ma Z., Zhao Q. (2012). Dissolution and regeneration of collagen fibers using ionic liquid. Int. J. Biol. Macromol..

[60] Rabotyagova O.S., Cebe P., Kaplan D.L. (2008). Collagen structural hierarchy and susceptibility to degradation by ultraviolet radiation. Mater. Sci. Eng. C Mater. Biol. Appl..

[61] Tronci G., Doyle A., Russell S.J., Wood D.J. (2012). Structure-property-function relationships in triple-helical collagen hydrogels. MRS Proc..

[62] Ribeiro A.R., Barbaglio A., Oliveira M.J., Santos R., Coelho A.V., Ribeiro C.C., Wilkie I.C., Carnevali M.D.C., Barbosa M.A. (2012). Correlations Between the Biochemistry and Mechanical States of a Sea-Urchin Ligament: A Mutable Collagenous Structure. Biointerphases.

[63] Nagai T., Suzuki N., Tanoue Y., Kai N., Nagashima T. (2010). Characterization of acid-soluble collagen from skins of surf smelt (*Hypomesus pretiosus japonicus Brevoort*). Food Nutr. Sci..

[64] Palpandi C., Ramasamy P., Rajinikanth T., Vairamani S., Shanmugam A. (2010). Extraction of collagen from mangrove Archeaogastropod *Nerita (Dostia) crepidularia* Lamarck, 1822. Am.-Euras. J. Sci. Res..

[65] Belbachir K., Noreen R., Gouspillou G., Petibois C. (2009). Collagen types analysis and differentiation by FTIR spectroscopy. Anal. Bioanal. Chem..

[66] Doyle B.B., Bendit E.G., Blout E.R. (1975). Infrared spectroscopy of collagen and collagen-like polypeptides. Biopolymers.

[67] Kaminska A., Sionkowska A. (1996). Effect of UV radiation on the infrared spectra of collagen. Polym. Degrad. Stabil..

[68] Kong J., Yu S. (2007). Fourier transform infrared spectroscopic analysis of protein secondary structures. Acta Biochim. Biophys. Sin. Shanghai.

[69] Lee S.-H., Mirkin N.G., Krimm S. (1999). A quantitative anharmonic analysis of the amide A band in α-helical poly(L-Alanine). Biopolymers.

[70] Serdyuk I.N., Zaccai N.R., Zaccai J. (2007). Methods in Molecular Biophysics. Structure, Dynamics, Function.

[71] Surewicz W.K., Mantsch H.H. (1988). New insight into protein secondary structure from resolution-enhanced infrared spectra. Biochim. Biophys. Acta BBA Protein Struct. Mol. Enzym..

[72] Guilbert M., Said G., Happillon T., Untereiner V., Garnotel R., Jeannesson P., Sockalingum G.D. (2013). Probing non-enzymatic glycation of type I collagen: A novel approach using Raman and infrared biophotonic methods. Biochim. Biophys. Acta BBA Protein Struct. Mol. Enzym..

[73] Derrick M. (1991). Evaluation of the state of degradation of Dead Sea Scroll samples using FT-IR spectroscopy. Book Pap. Group Annu..

[74] Wary R., Sivaraj S., Karthikeyan G., Pathak R.K., Suraj S.L.M., Dasararaju G., Kannayiram G. (2014). Chitosan gallic acid microsphere incorporated collagen matrix for chronic wounds: Biophysical and biochemical characterization. Int. J. Pharm. Pharm. Sci..

[75] Goissis G., Piccirili L., Goes J.C., Plepis A., Das-Gupta D.K. (1998). Anionic Collagen: Polymer Composites with Improved Dielectric and Rheological Properties. Artif. Organs.

[76] Plepis A.M.D.G., Goissis G., Das-Gupta D.K. (1996). Dielectric and pyroelectric characterization of anionic and native collagen. Polym. Eng. Sci..

[77] Smith B. (1999). Infrared Spectral Interpretation. A Systematic Approach.

[78] Petibois C., Gouspillou G., Wehbe K., Delage J.-P., Déléris G. (2006). Analysis of type I and IV collagens by FT-IR spectroscopy and imaging for a molecular investigation of skeletal muscle connective tissue. Anal. Bioanal. Chem..

[79] Gunasekaran S., Natarajan R.K., Renganayaki V., Natarajan S. (2006). Vibrational spectra and thermodynamic analysis of metformin. Indian J. Pure Appl. Phys..

[80] Doillon C.J., Silver F.H. (1986). Collagen-based wound dressing: Effects of hyaluronic acid and fibronectin on wound healing. Biomaterials.

[81] Pieper J.S., Oosterhof A., Dijkstra P.J., Veerkamp J.H., van Kuppevelt T.H. (1999). Preparation and characterization of porous crosslinked collagenous matrices containing bioavailable chondroitin sulphate. Biomaterials.

[82] Doillon C.J., Whyne C.F., Berg R.A., Olson R.M., Silver F.H. (1984). Fibroblast-collagen sponge interactions and the spatial deposition of newly synthesized collagen fibers in vitro and in vivo. Scan. Electron Microsc..

[83] Pal K., Banthia A.K., Majumdar D.K. (2009). Polymeric hydrogels: Characterization and biomedical applications. Des. Monomers Polym..

[84] Yoshida Z., Oda R. (1964). Intermolecular hydrogen bond involving a π-base as the proton acceptor. I. Detection by the refractive index method. J. Phys. Chem..

[85] Blackburn R.S., Burkinshaw S.M. (2003). Treatment of cellulose with cationic, nucleophilic polymers to enable reactive dyeing at neutral pH without electrolyte addition. J. Appl. Polym. Sci..

[86] Blackburn R.S., Harvey A., Kettle L.L., Manian A.P., Payne J.D., Russell S.J. (2007). Sorption of Chlorhexidine on Cellulose: Mechanism of Binding and Molecular Recognition. J. Phys. Chem. B.

[87] Flory P.J. (1971). Principles of Polymer Chemistry.

[88] Bray J.K., Merrill E.W. (1973). Poly(vinyl alcohol) hydrogels. Formation by electron beam irradiation of aqueous solutions and subsequent crystallization. J. App. Polym. Sci..

[89] Ofner C.M., Bubnis W.A. (1996). Chemical and swelling evaluations of amino group crosslinking in gelatin and modified gelatin matrices. Pharm. Res..

[90] Bohidar H.B., Jena S.S. (1993). Kinetics of sol–gel transition in thermoreversible gelation of gelatin. J. Chem. Phys..

[91] Tanioka A., Tazawa T., Miyasaka K., Ishikawa K. (1974). Effects of water on the mechanical properties of gelatin films. Biopolymers.

[92] Tanford C. (1961). Physical Chemistry of Macromolecules.

[93] Nelson D.L., Cox M.M. (2004). Lehninger Principles of Biochemistry.

[94] Stoll V.S., Blanchard J.S., Abelson J.N., Simon M.I. (2009). Buffers: Principles and practice. Methods in Enzymology. Guide to Protein Purification.

[95] Davies C.W. (1938). The extent of dissociation of salt in water. Part VIII. An equation for the mean ionic activity coefficient of an electrolyte in water, and a revision of the dissociation constants of some sulphates. J. Chem. Soc..

[96] Davies C.W. (1962). Ion Association.

[97] Ritsema C.J. (1993). Estimation of activity coefficients of individual ions in solutions with ionic strengths up to 0.3 mol dm^−3^. J. Soil. Sci..

[98] Fietzek P.P., Kuhn K., Hall D.A., Jackson D.S. (1976). The Primary Structure of Collagen. International Review of Connective Tissue Research.

[99] Mokrousova O.R., Volfkovich Y.M., Volfkovich Y.M., Filoppov A.N., Bagotsky V.S. (2014). Hide and Skin of Mammals. Structural Properties of Porous Materials and Powders Used in Different Fields of Science and Technology.

[100] Muiznieks L.D., Keeley F.W. (2013). Molecular assembly and mechanical properties of the extracellular matrix: A fibrous protein perspective. Biochim. Biophys. Acta BBA Mol. Basis Dis..

[101] Shoulders M.D., Raines R.T. (2009). Collagen structure and stability. Annu. Rev. Biochem..

[102] Ventre M., Padovani M., Cavington A.D., Netti P.A. Composition, Structure and Physical Properties of Fetal Calf Skin. Proceedings of the IULTCS Eurocongress 2006.

[103] Brosse J., Sabatier B. (2005). Use of Superabsorbent Polymers for Treating Raw Skins, Corresponding Compositions and Methods and Resulting Treated Skins. U.S. Patent.

[104] Manicourt D.-H., Devogelaer J.-P., Thonar E.J.-M.A., Seibels M.J., Robins S.P., Bilezikian J.P. (2006). Products of Cartilage Metabolism. Dynamics of Bone and Cartilage Metabolism.

[105] Kuhn K., Mayne R., Burgeson R.E. (1987). The Classical Collagens: Type I., II, and III. Structure and Function of Collagen Types.

[106] Cawston T.E., Murphy G. (1981). [52] Mammalian collagenases. Methods Enzymol..

[107] Welgus H.G., Jeffrey J.J., Stricklin G.P., Roswit W.T., Eisen A.Z. (1980). Characteristics of the action of human skin fibroblast collagenase on fibrillar collagen. J. Biol. Chem..

[108] Grassi M., Grassi G., Lapasin R., Colombo I. (2007). Understanding Drug Release and Absorption Mechanisms. A Physical and Mathematical Approach.

[109] Boateng J.S., Matthews K.H., Stevens H.N., Eccleston G.M. (2008). Wound Healing Dressings and Drug Delivery Systems: A Review. J. Pharm. Sci..

[110] Wu N., Wang L.-S., Tan D.C.-W., Moochhala S.M., Yang Y.-Y. (2005). Mathematical modeling and in vitro study of controlled drug release via a highly swellable and dissoluble polymer matrix: Polyethylene oxide with high molecular weights. J. Control. Release.

[111] Brazel C.S., Peppas N.A. (2000). Modeling of drug release from swellable polymers. Eur. J. Pharm. Biopharm..

[112] Peppas N.A., Bures P., Leobandung W., Ichikawa H. (2000). Hydrogels in pharmaceutical formulations. Eur. J. Pharm. Biopharm..

[113] Grassi M., Lapasn R., Pricl S. (1998). Modeling of Drug Release from a Swellable Matrix. Chem. Eng. Commun..

[114] Morawetz H. (1996). Nature of the hypercoiled form of poly(methacrylic acid) in water at low pH. Macromolecules.

[115] Kalyanasundaram K., Thomas J.K. (1977). Environmental effects on vibronic band intensities in pyrene monomer fluorescence and their application in studies of micellar systems. J. Am. Chem. Soc..

[116] Winnik F.M., Regismond S.T.A., Kwak J.K.T. (1998). Fluorescence methods in the study of polymer-surfactant systems. Polymer-Surfactant Systems.

[117] Zana R., Lévy H., Kwetkat K. (1998). Mixed micellization of dimeric (gemini) surfactants and conventional surfactants. I. Mixtures of an anionic dimeric surfactant and of the nonionic surfactants C12E5and C12E8. J. Colloid Interface Sci..

[118] Regev O., Zana R. (1999). Aggregation behavior of Tyloxapol, a nonionic surfactant oligomer, in aqueous solution. J. Colloid Interface Sci..

[119] Micutz M., Staicu T., Leca M. (2005). Fluorescence and electron microscopy studies on collagen type I-ionic surfactants systems with gel consistency. Rev. Roum. Chim..

[120] Holmberg K., Jonsson B., Kronberg B., Lindman B. (2004). Surfactants and Polymers in Aqueous Solution.

[121] Rosen M.J. (2004). Surfactants and Interfacial Phenomena.

[122] Adane L., Bharatam P.V. (2008). Tautomeric preferences and electron delocalization in biurets, thiobiurets, and dithiobiurets: An *ab initio* study. Int. J. Quantum Chem..

[123] Bharatam P.V., Patel D.S., Iqbal P. (2005). Pharmacophiric features of biguanide derivatives: An electronic and structural analysis. J. Med. Chem..

[124] Ernst S.R., Cagle F.W. (1977). Biguanide. Acta Cryst..

[125] Maksić Z.B., Kovačević B. (2000). Absolute Proton Affinity of Some Polyguanides. J. Org. Chem..

[126] Ernst S.R. (1977). Biguanide hydrochloride. Acta Crystallogr. Sect. B Struct. Crystallogr. Cryst. Chem..

[127] Mason P.E., Dempsey C.E., Vrbka L., Heyda J., Brady J.W., Jungwirth P. (2009). Specificity of ion-protein interactions: Complementary and competitive effects of tetrapropylammonium, guanidinium, sulfate, and chloride ions. J. Phys. Chem. B.

[128] Liu X., Wu X. (2015). Flurescence enhancement of Fisetin by silver nanoparticles with cetyltrimethyl ammonium bromide micelles. RSC. Adv..

[129] Kuperkar K.C., Mata J.P., Bahadur P. (2011). Effect of 1-alkanols/salt on the cationic surfactant micellar aqueous solution—A dynamic light scattering study. Colloids Surf. A Physicochem. Eng. Aspects.

[130] Dorshow R., Briggs J., Bunton C.A., Nicoli D.F. (1982). Dynamic light scattering from cetyltrimethylammonium bromide micelles. Intermicellar interactions at low ionic strengths. J. Phys. Chem..

[131] Van Oosten B., Marquardt D., Komljenović I., Bradshaw J.P., Sternin E., Harroun T.A. (2014). Small molecule interaction with lipid bilayers: A molecular dynamics study of chlorhexidine. J. Mol. Graph. Model..

[132] Komljenović I., Marquardt D., Harroun T., Sternin E. (2010). Location of chlorhexidine in DMPC model membranes: A neutron diffraction study. Chem. Phys. Lipids.

[133] Chen Y., Flanagan D., Qiu Y., Chen Y., Zhang G.G.Z., Liu L., Porter W.R. (2009). Theory of Diffusion and Pharmaceutical applications. Developing Solid Oral Dosage Forms. Pharmaceutical Theories and Practice.

[134] Peppas N.A., Brannon-Peppas L. (1994). Water diffusion and sorption in amorphous macromolecular systems and foods. J. Food Eng..

[135] Crank J. (1975). The Mathematics of Diffusion.

[136] Enscore D., Hopfenberg H., Stannett V. (1977). Effect of particle size on the mechanism controlling n-hexane sorption in glassy polystyrene microspheres. Polymer.

[137] Ritger P.L., Peppas N.A. (1987). A simple equation for description of solute release I. Fickian and non-fickian release from non-swellable devices in the form of slabs, spheres, cylinders or discs. J. Control. Release.

[138] Korsmayer R.W., Peppas N.A. (1984). Solute and penetrant diffusion in swellable polymers. III. Drug release from glassy poly(HEMA-co-NVP) copolymers. J. Control. Release.

[139] Siepmann J., Peppas N.A. (2012). Modeling of drug release from delivery systems based on hydroxypropyl methylcellulose (HPMC). Adv. Drug Deliv. Rev..

[140] Brazdaru L., Micutz M., Staicu T., Albu M.G., Sulea D., Leca M. (2015). Structural and rheological properties of collagen hydrogels containing tannic acid and chlorhexidine digluconate intended for topical applications. Comptes Rendus Chim..

[141] Ghigo G., Berto S., Minella M., Vione D., Alladio E., Nurchi V.M., Lachowicz J., Daniele P.G. (2018). New insights into the protogenic and spectroscopic properties of commercial tannic acid. The role of gallic acid impurities. N. J. Chem..

[142] Simoes M.C., Hughes K.J., Ingham D.B., Ma L., Pourkashanian M. (2017). Estimation of the thermochemical radii and ionic volumes of complex ions. Inorg. Chem..

[143] Kunz W. (2010). Specific ion effects in colloidal and biological systems. Curr. Opin. Colloid Interface Sci..

[144] Collins K. (2004). Ions from the Hofmeister series and osmolytes: Effects on proteins in solution and in the crystallization process. Methods.

[145] Micutz M., Circu V., Ilis M., Staicu T. (2022). Novel Gemini Surfactant for Binding Eu(III)-Polyoxometalate into Hydrogels and Polymer Latexes. Gels.

