# Supplementary material for: 3D Porous Collagen Matrices—A Reservoir for In Vitro Simultaneous Release of Tannic Acid and Chlorhexidine
Source: Pharmaceutics. 2022 Dec 26;15(1):76. doi: 10.3390/pharmaceutics15010076 (PMC9865545; doi:10.3390/pharmaceutics15010076)
Supplement: Supplementary file 1 [file pharmaceutics-15-00076-s001.zip › pharmaceutics-2092853-supplementary.pdf]

Supplementary Materials

3D Porous Collagen Matrices—A Reservoir for In Vitro Simultaneous Release of Tannic Acid and Chlorhexidine

Lavinia Brăzdaru [1] Teodora Staicu [1,*] Mădălina Georgiana Albu Kaya [2] Ciprian Chelaru [2] Corneliu Ghica [3] Viorel Cîrcu [4] Minodora Leca [1] Mihaela Violeta Ghica [5] and Marin Micutz [1,6,*]

[1] *Department of Physical Chemistry, University of Bucharest, 4-12 Regina Elisabeta Blvd., 030018 Bucharest, Romania*

[2] *Leather and Footwear Research Institute, 93 Ion Mincu St., 031215 Bucharest, Romania*

[3] *National Institute of Materials Physics, 105 bis Atomistilor St., 077125 Magurele, Romania*

[4] *Department of Inorganic Chemistry, University of Bucharest, 4-12 Regina Elisabeta Blvd., 030018 Bucharest, Romania*

[5] *Faculty of Pharmacy, University of Medicine and Pharmacy "Carol Davila", 6 Traian Vuia St., 020956 Bucharest, Romania*

[6] *Institute of Physical Chemistry "Ilie Murgulescu", Romanian Academy, 202 Spl. Independenţei, 060021 Bucharest, Romania*

* *Correspondence: teos@gw-chimie.math.unibuc.ro (T.S.); micutz@gw-chimie.math.unibuc.ro (M.M.)*

Content

1. Table S1. Characteristic infrared bands for some collagen-based matrices…………..…. p. 3

2. Table S2. The amino acid residues and their features mainly involved in generating the fixed electrical charges borne by a single triple helix of collagen type I extracted from calf skin……………………………………………………………………………………….. p. 3

3. Table S3. Planar and spatial structures of some extended and folded conformers/tautomers of chlorhexidine dication, highlighting the distances between chlorine atoms………………………………………………………………………………. pp. 4-6

4. Table S4. The values matrix for the TA/CHDG compositions used to guide the experimental measurements. The values inside the nonbolded cells are the ratios of TA to CHDG concentrations..…………………………………………………….………………….. p. 7

5. Table S5. The values of the kinetic constants (k), diffusional exponents (n), coefficients of determination (R^2) and fractional releases after 11 h (c_{11}) for the entirely (up to 100%) determined TA release from the TA-collagen matrices..…………………………………… p. 7

6. Table S6. The values of the kinetic constants (k), diffusional exponents (n), coefficients of determination (R^2) and fractional releases after 11 h (c_{11}) for the entirely (up to 100%) determined TA release from the TA-CHD-collagen matrices……….…………………... p. 7

7. Table S7. The values of the kinetic constants (k), diffusional exponents (n), coefficients of determination (R^2) and fractional releases after 11 h (c_{11}) for the entirely (up to 100%) determined CHDG release from the TA-CHD-collagen matrices………………..……… p. 8

8. Table S8. The values of the kinetic constants (k), diffusional exponents (n), coefficients of determination (R^2) and weights of (a) Fickian and (b) non-Fickian-Case II diffusion for the TA release (up to 0.6) from the TA-collagen matrices………………..…………….... p. 8

9. Table S9. The values of the kinetic constants (k), diffusional exponents (n), coefficients of determination (R^2) and weights of (a) Fickian and (b) non-Fickian-Case II diffusion for the TA release (up to 0.6) from the TA-CHD-collagen matrices…………………….….. p. 8

10. Table S10. The values of the kinetic constants (k), diffusional exponents (n), coefficients of determination (R^2) and weights of (a) Fickian and (b) non-Fickian-Case II diffusion for the CHDG release (up to 0.6) from the TA-CHD-collagen matrices………... p. 9

11. Figure S1. FT-IR spectra (extended and the fingerprint region) for the collagen-based matrices with the specified compositions (CHDG-free and 9.09% CHDG)..……………… p. 9

12. Figure S2. FT-IR spectra (extended and the fingerprint region) for the collagen-based matrices with the specified compositions (4.55% CHDG)…………………………. p. 9

13. Figure S3. FT-IR spectra (extended and the fingerprint region) for the collagen-based matrices with the specified compositions (1.82% CHDG)………………………………… p. 10
14. Figure S4. SEM micrographs (taken from sections parallel to air-sided face) for (a) collagen matrix and collage-based matrices with (b) TA(10%) and with (c) TA(10%)-CHDG(9.09%)…………………………………………………………………………... p. 10
15. Figure S5. Estimated temperature evolution of buffer density (upper graph) obeying the same tendency as for water density (bottom plot) (buffer density of 1.098 g/cm^3 at 20^{0}C was pycnometrically determined) (see www.vip-ltd.co.uk for water density values; accessed on November 19, 2022)……………………………………………………... p. 10
16. Figure S6. CHDG release from the collagen-based matrices with (a) 5% TA, (b) 10% TA and (c) 15% TA into PBS-HTAB solution (pH 7.4) at 37^{0}C…………………………… p. 11
17. Determination of cmc of HTAB in 10 mM sodium buffer phosphate at 37^{0}C by using I_1/I_3 ratio of pyrene (Figure S7. Concentration dependence of I_1/I_3 of Py in HTAB solution at 37^{0}C (5x10^{-7}M Py in 10 mM buffer phosphate))………………………………….... pp. 11-12
18. Figure S8. Changes in UV-VIS spectra of TA in different aqueous environments (a-d), the corresponding spectra of TA and CHDG (taken separately) in PBS and HTAB (e) and the UV-VIS spectra for three TA-CHDG mixtures in the same release solution (PBS+HTAB) at the specified compositions (f)…………………………………………. p. 12
19. Experimental algorithm on determination of TA/CHDG concentrations in release medium (Figure S9. (a) Obeying Lambert-Beer's law starting from (b) an initial solution of minimum concentration of 2.03×10^{-4}% TA and 2.9×10^{-4}% CHDG and a final solution of maximum concentration of 1.20×10^{-3}% TA and 1.71×10^{-3}% CHDG in PBS and HTAB with keeping the concentration ratio constant (TA/CHDG – 0.7; the arrow indicates how components concentrations increase))……………………………………………….... p. 13
20. Figure S10. TA (filled symbols) and CHDG (open symbols) release from collagen-based matrices into PBS-HTAB solution (pH 7.4) at 37^{0}C……………………………… p. 13
21. Ionic strength and temperature effects on practical/apparent acid exponent pK_{ap}……… pp. 14-15
22. Relationships used for defining some quantities at equilibrium swelling required in the evaluation of crosslinking degree associated with the studied collagen-based matrices…… p. 15-16

(1) Table S1. Characteristic infrared bands for some collagen-based matrices.

Designation	Collagen	Collagen TA–5% CHDG-1.82%	Collagen TA–10% CHDG-1.82%	Collagen TA–15% CHDG-1.82%	Collagen TA–5% CHDG-4.55%	Collagen TA–10% CHDG-4.55%	Collagen TA–15% CHDG-4.55%	Assignment
-	3455	3465	3474	3474	3474	3460	3484	(hydrogen bond) hydrogen bonded water[60,67]
Amide A	3301	3294	3296	3292	3299	3298	3299	NH stretching[64,68-70]/C=N (guanidine) symmetrical stretching[68]
Amide B	3077	3077	3080	3079	3084	3080	3080	NH stretching[64,68-70]
-	2933	2935	2938	2936	2933	2938	2937	CH_2 asymmetrical stretching[60,64]
-	2873	2875	2882	2882	2875	2880	2875	CH_2 symmetrical stretching[64] CH_3 asymmetrical stretching[60]
Amide I	1630	1629	1631	1630	1631	1631	1631	C=O stretching[64,68-70]
Amide II	1545	1540	1540	1539	1538	1538	1537	CN stretching, NH bending[64,68-70]
-	1450	1448	1448	1447	1447	1448	1447	CH_2 bending[64]
-	1339	1336	1335	1334	1334	1336	1333	in plain OH (phenol) bending[77]
Amide III	1236	1236	1234	1233	1233	1235	1232	CN stretching, NH bending[64,68-70], CH_2 wagging[61,66,78], CN stretching (guanidine)[79]
-	1204	1203	1202	1201	1201	1202	1200	C-O stretching (C-OH in phenol)[77]
-	1080	1080	1080	1079	1080	1080	1080	C-O stretching (carbohydrate moiety)[64,65,72,78]/ C-O-C asymmetrical stretching
-	1033	1033	1033	1033	1033	1033	1033	C-O stretching (carbohydrate moiety)[64,65,72,78]/ C-O-C symmetrical stretching
ν_{amideI}-$\nu_{amideII}$	85	89	91	91	93	93	94	
$A_{amide\ III}/A_{1450}$	0.9	0.9	0.9	0.9	0.90	0.9	0.9	

(2) Table S2. The amino acid residues and their features mainly involved in generating the fixed electrical charges borne by a single triple helix of collagen type I extracted from calf skin [4]

Amino acid	α1(I) chain	α2(I) chain	pK_a[93] (side group)	dpK_a/dt,[94]*** $1/^0C$
Asp	33	24	3.65 (3.56)	0.0002 (acetate; pK_a=4.76)
Glu	52	46	4.25 (4.16)	0.0002 (acetate; pK_a=4.76)
His	3	8	6.00 (5.85)	-0.020 (imidazole; pK_a=6.95)
Arg	53	56	12.48 (12.22)	-0.029 (ethanolamine; pK_a=9.50)
Lys	34	21	10.53 (10.27)	-0.029 (ethanolamine; pK_a=9.50)
Asn	13	23		
Gln	27	24		

Gly (N-terminus)	1	1	9.60 (9.34) $-NH_3^+/-NH_2$*	-0.029 (ethanolamine; pK_a=9.50)
Pro (C-terminus)	1	1	1.99 (1.90) -COOH/$-COO^-$**	0.0002 (acetate; pK_a=4.76)

*values counted for the three amino-terminal glycine residues per triple helix

**values counted for the three carboxy-terminal proline residues per every single triple helix (2α1(I)+ α2(I) chains)

***values considered by analogy to those specific to the buffers indicated in parentheses

(3) Table S3. Planar and spatial structures of some extended and folded conformers/tautomers of chlorhexidine dication, highlighting the distances between chlorine atoms.

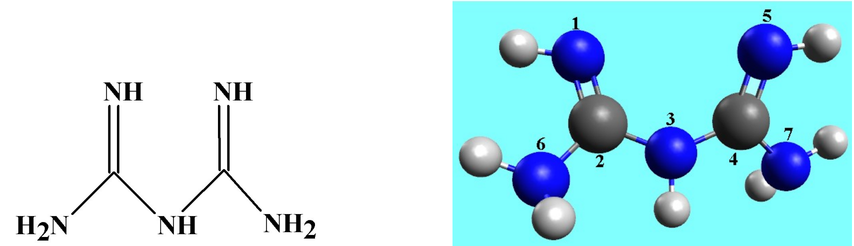

biguanide structure – old version

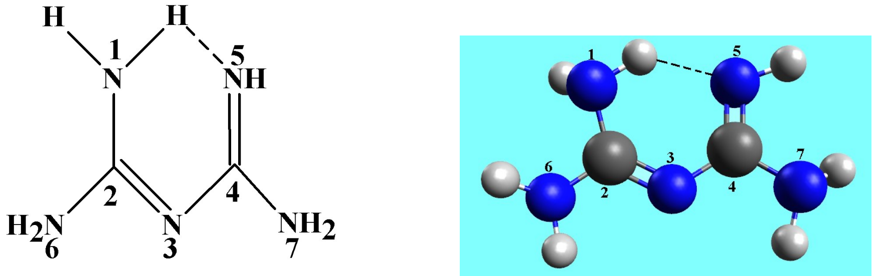

biguanide structure – actual ("right") version

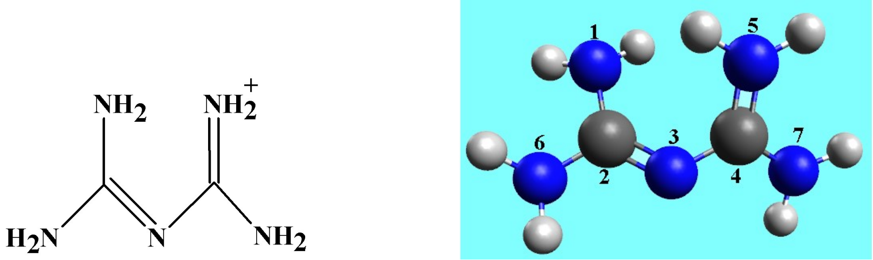

biguanidium cation

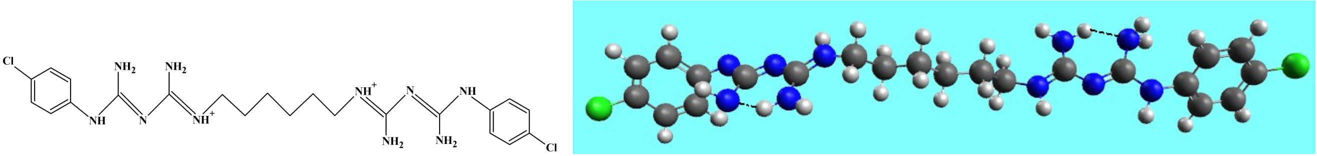

(1) extended conformation of chlorhexidine dication (CH^{++}); $d_{Cl\text{-}Cl}$= 29.0 Å

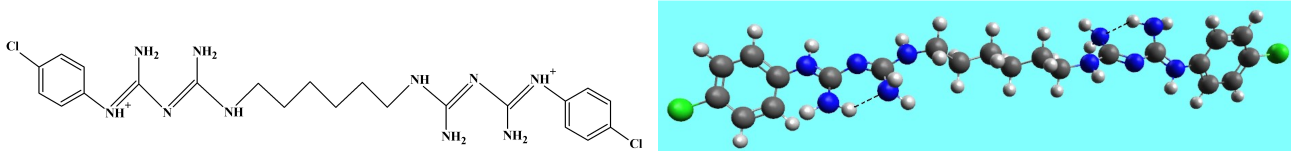

(2) extended conformation of CH^{++}; $d_{Cl\text{-}Cl}$= 29.1 Å

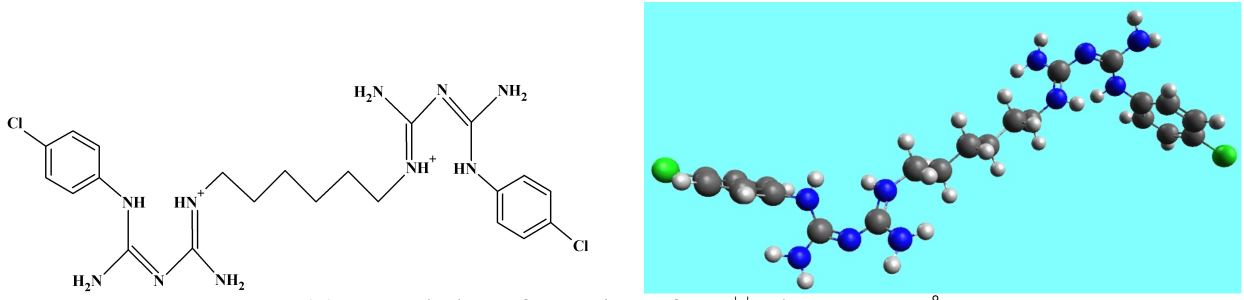

(3) extended conformation of CH^{++}; $d_{Cl\text{-}Cl}$= 24.3 Å

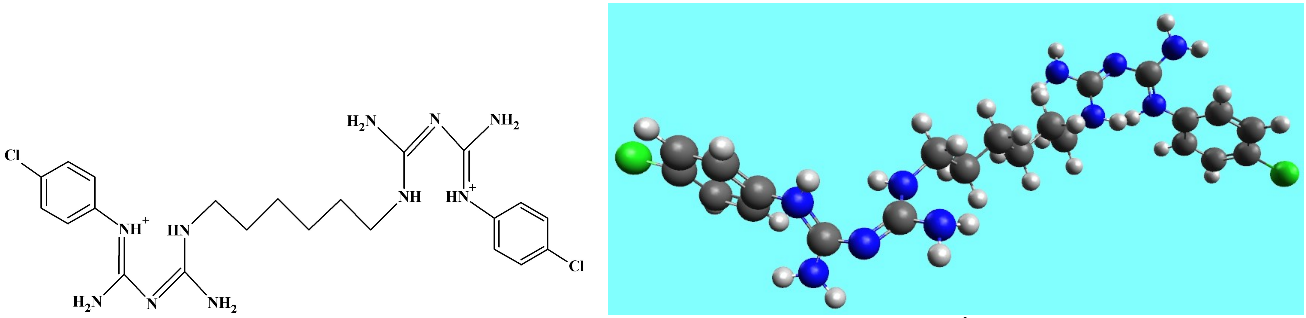

(4) extended conformation of CH^{++}; $d_{Cl\text{-}Cl}$= 23.8 Å

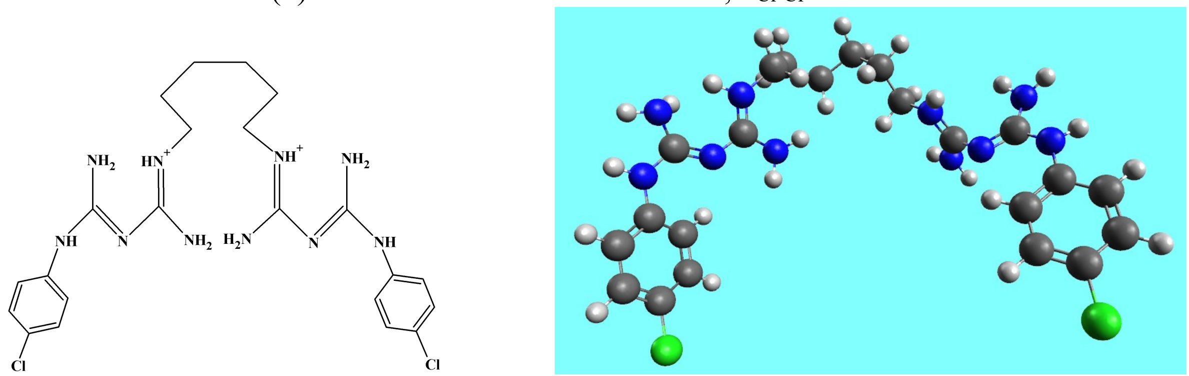

(5) folded conformation of CH^{++}; $d_{Cl\text{-}Cl}$= 13.9 Å

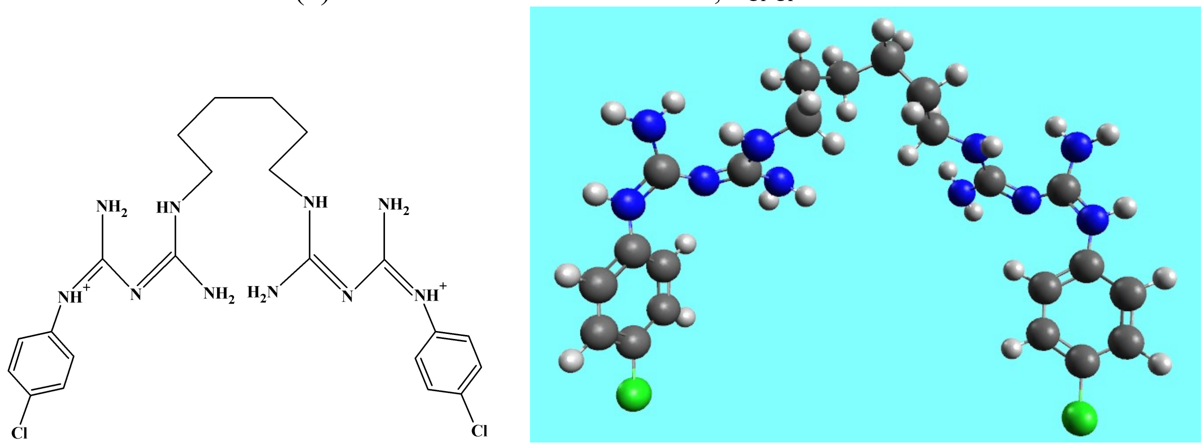

(6) folded conformation of CH^{++}; $d_{Cl\text{-}Cl}$= 13.4 Å

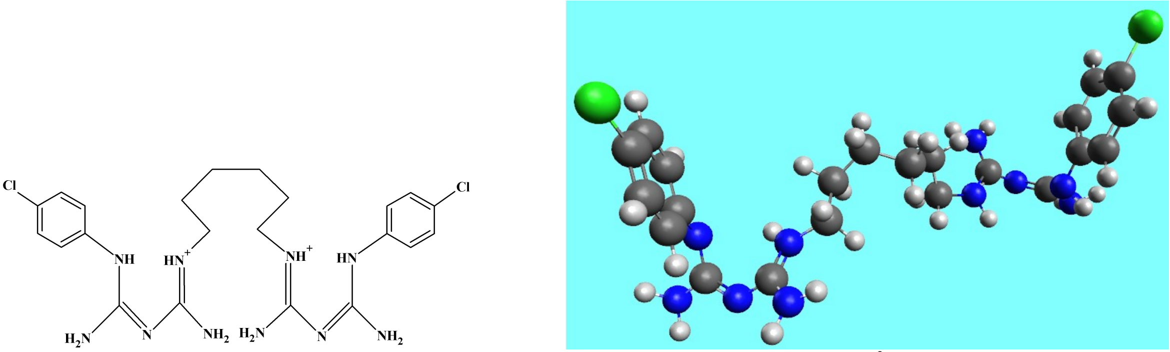
(7) folded conformation of CH^{++}; d_{Cl-Cl}= 15.9 Å

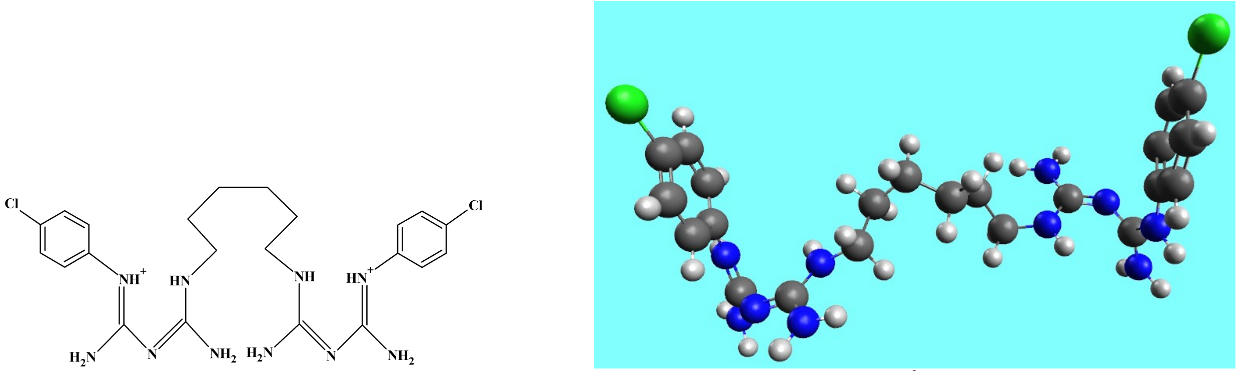
(8) folded conformation of CH^{++}; d_{Cl-Cl}= 15.5 Å

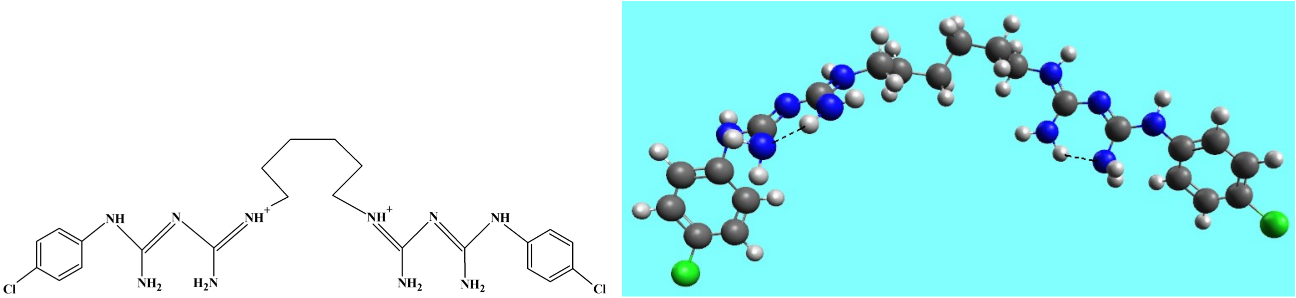
(9) folded conformation of CH^{++}; d_{Cl-Cl}= 22.7 Å

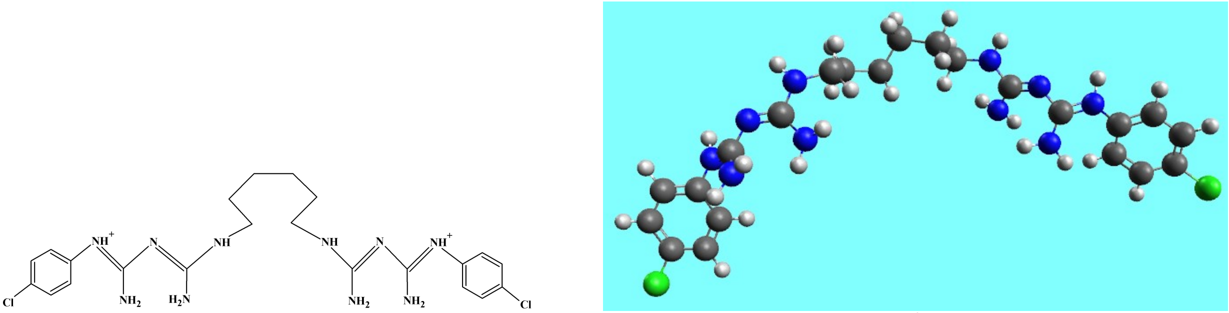
(10) folded conformation of CH^{++}; d_{Cl-Cl}= 22.8 Å

(4) Table S4. The values matrix for the TA/CHDG compositions used to guide the experimental measurements. The values inside the nonbolded cells are the ratios of TA to CHDG concentrations.

		**CHDG concentration x 10^4, %**						
		**2.53**	**5.07**	**7.60**	**10.13**	**12.67**	**15.20**	**17.73**
**TA concentration x 10^4, %**	**14.00**	5.53	2.76	1.84	1.38	1.11	0.92	0.79
	**12.00**	4.74	2.37	1.58	1.18	0.95	0.79	0.68
	**10.00**	3.95	1.97	1.32	0.99	0.79	0.66	0.56
	**8.00**	3.16	1.58	1.05	0.79	0.63	0.53	0.45
	**6.00**	2.37	1.18	0.79	0.59	0.47	0.39	0.34
	**4.00**	1.58	0.79	0.53	0.39	0.32	0.26	0.23
	**2.00**	0.79	0.39	0.26	0.20	0.16	0.13	0.11

(5) Table S5. The values of the kinetic constants (k), diffusional exponents (n), coefficients of determination (R^2) and fractional releases after 11 h (c_{11}) for the entirely (up to 100%) determined TA release from the TA-collagen matrices.

**% TA**	**kx10^2, min^{-n}**	**n**	**R^2**	**c_{11}, %**
**5**	0.712	0.735	0.99246	80
**10**	1.113	0.686	0.99168	90
**15**	0.962	0.699	0.99024	84

(6) Table S6. The values of the kinetic constants (k), diffusional exponents (n), coefficients of determination (R^2) and fractional releases after 11 h (c_{11}) for the entirely (up to 100%) determined TA release from the TA-CHD-collagen matrices.

**% TA**	**kx10^2, min^{-n}**	**n**	**R^2**	**c_{11}, %**
**CHDG – 0%**				
**5**	0.712	0.735	0.99246	80
**10**	1.113	0.686	0.99168	90
**15**	0.962	0.699	0.99024	84
**CHDG – 1.82%**				
**5**	2.549	0.562	0.99743	94
**10**	1.406	0.630	0.99578	81
**15**	1.041	0.628	0.99637	60
**CHDG – 4.55%**				
**5**	1.790	0.601	0.99597	85
**10**	0.863	0.689	0.99681	73
**15**	1.435	0.602	0.99590	68
**CHDG – 9.09%**				
**5**	1.290	0.619	0.99736	69
**10**	1.879	0.600	0.99772	90
**15**	2.003	0.577	0.99761	82

(7) Table S7. The values of the kinetic constants (k), diffusional exponents (n), coefficients of determination (R^2) and fractional releases after 11 h (c_{11}) for the entirely (up to 100%) determined CHDG release from the TA-CHD-collagen matrices.

% CHDG	kx10^2, min^{-n}	n	R^2	c_{11}, %
TA – 5%				
**1.82**	1.062	0.611	0.99554	54
**4.55**	1.038	0.611	0.99554	53
**9.09**	0.653	0.662	0.99431	46
TA – 10%				
**1.82**	1.048	0.616	0.99476	56
**4.55**	1.094	0.600	0.99436	51
**9.09**	0.836	0.619	0.99391	45
TA – 15%				
**1.82**	1.149	0.603	0.99735	56
**4.55**	1.149	0.603	0.99735	56
**9.09**	0.982	0.590	0.99198	43

The quantities k and n are related to the characteristics of a matrix and the swelling solvent (k) and transport/diffusion mechanism of AT/CHDG from the swollen matrix (n) according to Peppas-type relationship (see the paper text).

(8) Table S8. The values of the kinetic constants (k), diffusional exponents (n), coefficients of determination (R^2) and weights of (a) Fickian and (b) non-Fickian-Case II diffusion for the TA release (up to 0.6) from the TA-collagen matrices.

	According to eq. (15) (see paper text)			According to eq. (16) (see paper text)		
% TA	kx10^2, min^{-n}	n	R^2	a/relative weight, %	b/relative weight, %	R^2
**5**	0.396	0.846	0.99872	0.0416/3.2	1.2466/96.8	0.99997
**10**	0.643	0.792	0.99931	0.1119/7.7	1.3439/92.3	0.99985
**15**	0.511	0.821	0.99923	0.0732/5.2	1.3321/94.8	0.99992

(9) Table S9. The values of the kinetic constants (k), diffusional exponents (n), coefficients of determination (R^2) and weights of (a) Fickian and (b) non-Fickian-Case II diffusion for the TA release (up to 0.6) from the TA-CHD-collagen matrices.

	According to eq. (15)			According to eq. (16)		
% TA	kx10^2, min^{-n}	n	R^2	a/relative weight, %	b/relative weight, %	R^2
CHDG – 0%						
**5**	0.396	0.846	0.99872	0.0416/3.2	1.2466/96.8	0.99997
**10**	0.643	0.792	0.99931	0.1119/7.7	1.3439/92.3	0.99985
**15**	0.511	0.821	0.99923	0.0732/5.2	1.3321/94.8	0.99992
CHDG – 1.82%						
**5**	2.147	0.595	0.99957	0.4305/36.0	0.7641/64.0	0.99949
**10**	1.125	0.673	0.99808	0.2560/23.7	0.8291/76.4	0.99952
**15**	1.041	0.628	0.99637	0.2520/37.2	0.4250/62.8	0.99947
CHDG – 4.55%						
**5**	1.348	0.657	0.99935	0.2961/25.3	0.8739/74.7	0.99949
**10**	0.680	0.735	0.99929	0.1589/15.8	0.8470/84.2	0.99968
**15**	1.164	0.642	0.99914	0.2713/31.2	0.5986/68.8	0.99948
CHDG – 9.09%						
**5**	1.089	0.652	0.99918	0.2571/29.5	0.6139/70.5	0.99949
**10**	1.536	0.640	0.99964	0.3301/27.9	0.8538/72.1	0.99948
**15**	1.680	0.612	0.99986	0.3576/34.7	0.6740/65.3	0.99947

(10) Table S10. The values of the kinetic constants (k), diffusional exponents (n), coefficients of determination (R^2) and weights of (a) Fickian and (b) non-Fickian-Case II diffusion for the <u>CHDG release (up to 0.6)</u> from the TA-CHD-collagen matrices.

	According to eq. (15)			According to eq. (17) (see the paper text)		
**% CHDG**	**kx10^2, min^{-n}**	**n**	**R^2**	**a/relative weight, %**	**b/relative weight, %**	**R^2**
			**TA – 5%**			
**1.82**	1.062	0.611	0.99554	0.4148/31.1	0.9188/68.9	0.99948
**4.55**	1.038	0.611	0.99554	0.4054/31.1	0.8981/68.9	0.99948
**9.09**	0.653	0.662	0.99431	0.2777/21.9	0.9932/78.1	0.99950
			**TA – 10%**			
**1.82**	1.048	0.616	0.99476	0.4134/30.1	0.9617/69.9	0.99947
**4.55**	1.094	0.600	0.99436	0.4174/33.5	0.8287/66.5	0.99949
**9.09**	0.836	0.619	0.99391	0.3317/29.5	0.7944/70.5	0.99947
			**TA – 15%**			
**1.82**	1.149	0.603	0.99735	0.4413/32.8	0.9029/67.2	0.99948
**4.55**	1.254	0.580	0.99183	0.4816/32.8	0.9855/67.2	0.99948
**9.09**	0.982	0.590	0.99198	0.3664/35.8	0.6562/64.2	0.99950

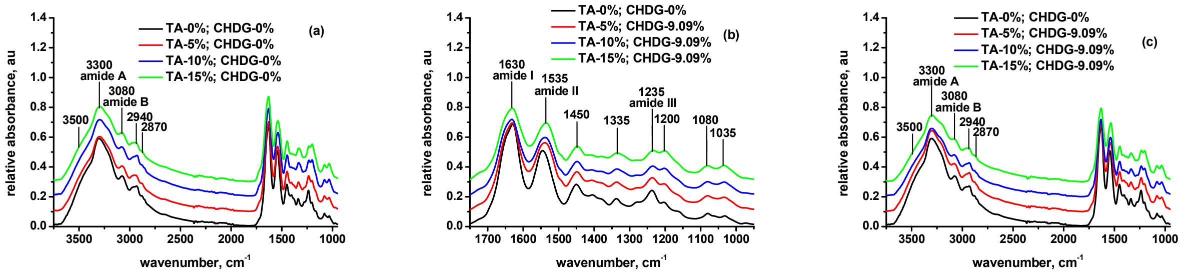

(11) Figure S1. FT-IR spectra (extended and the fingerprint region) for the collagen-based matrices with the specified compositions (CHDG-free and 9.09% CHDG).

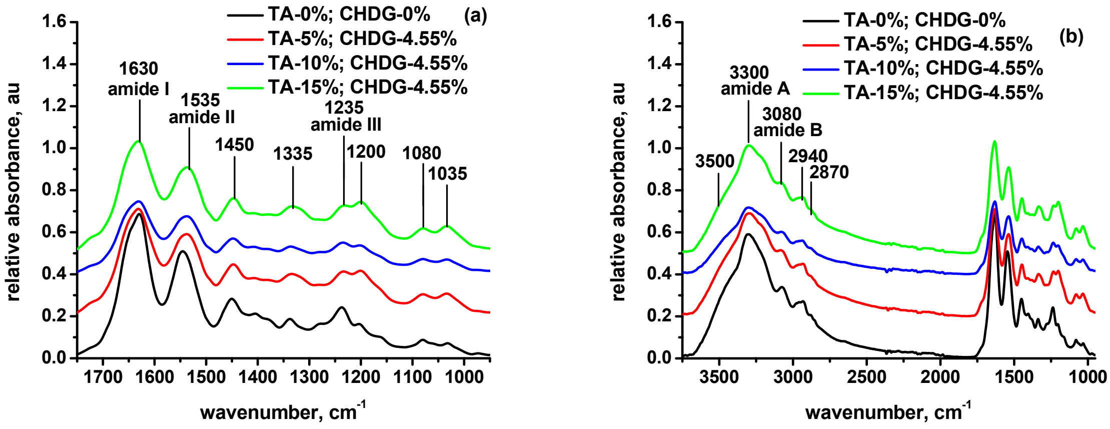

(12) Figure S2. FT-IR spectra (extended and the fingerprint region) for the collagen-based matrices with the specified compositions (4.55% CHDG).

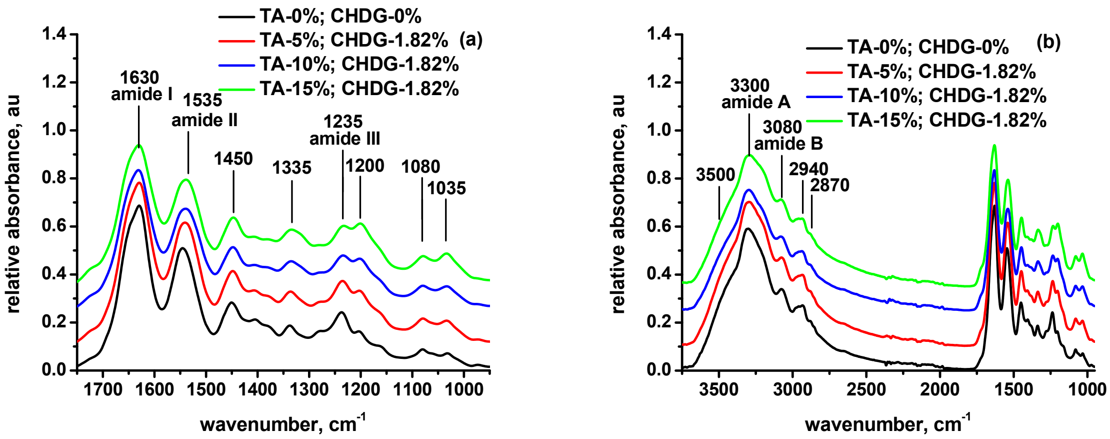

(13) **Figure S3. FT-IR spectra (extended and the fingerprint region) for the collagen-based matrices with the specified compositions (1.82% CHDG).**

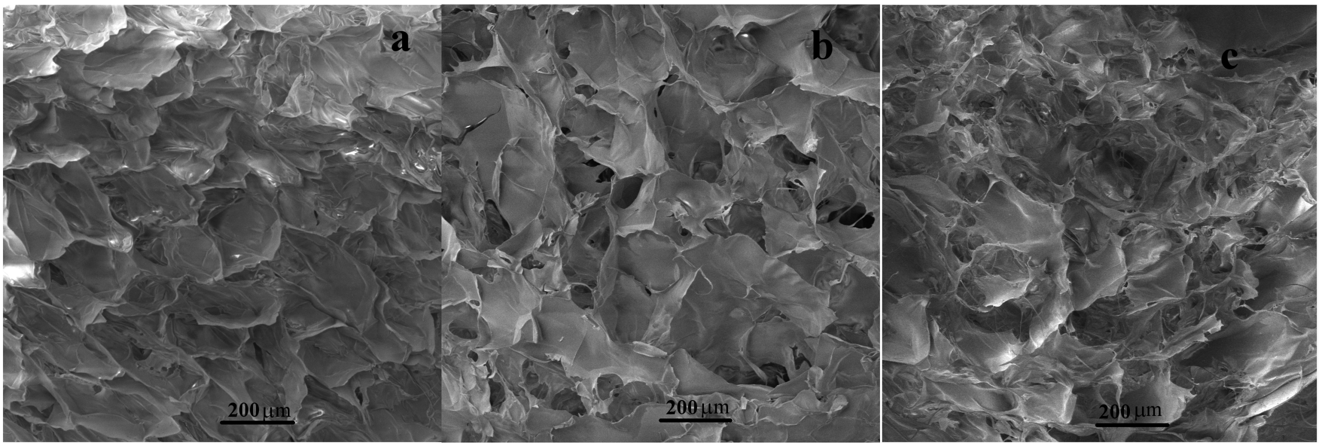

(14) **Figure S4. SEM micrographs (taken from sections parallel to air-sided face) for (a) collagen matrix and collage-based matrices with (b) TA(10%) and with (c) TA(10%)-CHDG(9.09%)**

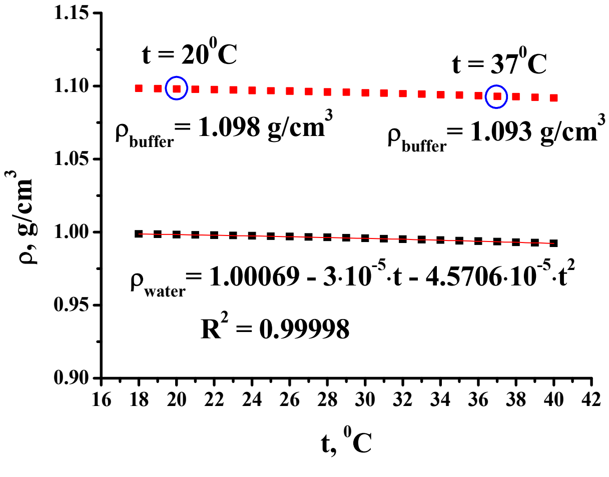

(15) **Figure S5. Estimated temperature evolution of buffer density (upper graph) obeying the same tendency as for water density (bottom plot) (buffer density of 1.098 g/cm^3 at 20^{0}C was pycnometrically determined) (see <u>www.vip-ltd.co.uk</u> for water density values; accessed on November 19, 2022).**

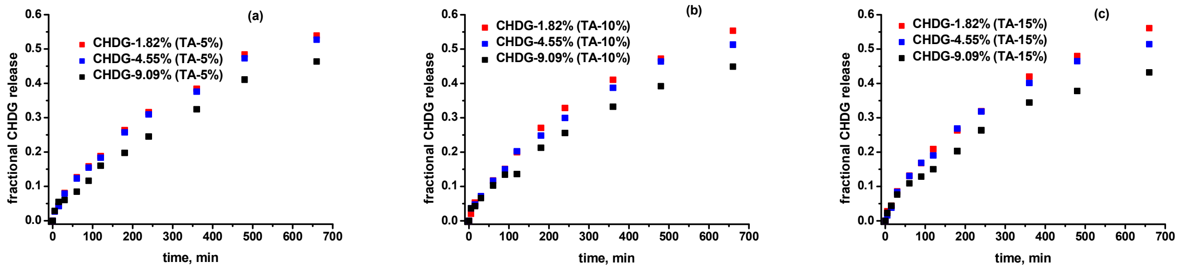

(16) Figure S6. CHDG release from the collagen-based matrices with (a) 5% TA, (b) 10% TA and (c) 15% TA into PBS-HTAB solution (pH 7.4) at 37^{0}C.

(17) Determination of *cmc* of HTAB in 10 mM sodium buffer phosphate at 37^{0}C by using I_1/I_3 ratio of pyrene

It is possible to determine critical micelle concentration (*cmc*) of a surfactant by using pyrene (Py) as fluorescence hydrophobic probe. This is based on the so-called I_1/I_3 rule of Py (I_1 and I_3 are the intensities of the of the 1st and 3rd vibronic bands located near 373 and 384 nm, respectively, in Py emission spectrum: excitation wavelength 334 nm, spectrum range 350-450 nm) according to that the solvent micropolarity sensed by Py is well correlated with value of I_1/I_3 ratio [114,115]. Thus, the lower polarity of the microenvironment around Py, the lower value of I_1/I_3 will be and vice-versa. Applying this to a surfactant solution, it was noticed a steeply decreasing in I_1/I_3 value during micelle formation that demonstrates Py solubilization preferentially within the hydrophobic microdomains generated by micellization [116]. According to Zana et al. [117,118], lower *cmc* values (generally below 10^{-2}M) should be determined by considering *cmc* as the abscissa of the inflection point of the steeply decreasing in I_1/I_3-surfactant concentration dependence (lin-log). The inflection point may be found by data fitting with a Boltzmann sigmoid function as shown elsewhere [119]:

$$\frac{I_1}{I_3}=\left(\frac{I_1}{I_3}\right)_{min}+\frac{\left(\frac{I_1}{I_3}\right)_{max}-\left(\frac{I_1}{I_3}\right)_{min}}{1+\exp\left(\frac{\log[HTAB]-\log(cmc)}{b}\right)} \qquad (S1)$$

where $(I_1/I_3)_{max}$ and $(I_1/I_3)_{min}$ are the upper and the bottom fitted values of I_1/I_3, log(*cmc*) – the inflection point and b – a parameter which describes the steepness of the sigmoid dependence.

Applying this function to fit the experimental data concerning HTAB, a value of 7.3x10^{-5} M resulted (Figure S7). It is an expected value of *cmc* whether the ionic strength effect of drastically diminishing *cmc* of an ionic surfactant is taken into account [120,121]. Indeed, *cmc* of HTAB in water was found to be 7.7x10^{-4} M, a little higher than that in NaOH solution of pH 8.0 at room temperature [58].

It is important to mention that all HTAB solutions were prepared by diluting an initial stock solution (in 10 mM sodium phosphate buffer, NaCl – 0.154 M, Py – ca. 10^{-6} M, pH 7.4) of HTAB with the same solvent consisting of sodium phosphate buffer and Py at 37^{0}C.

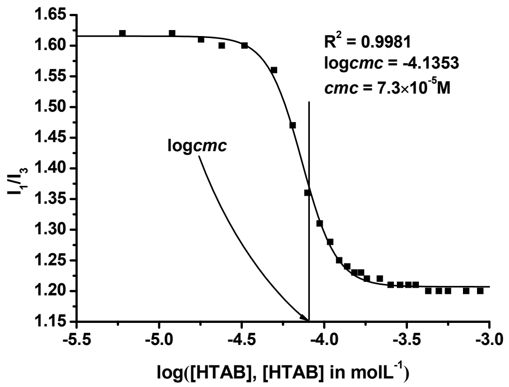

Figure S7. Concentration dependence of I_1/I_3 of Py in HTAB solution at 37^0C (10^{-6} M Py in PBS, pH 7.4).

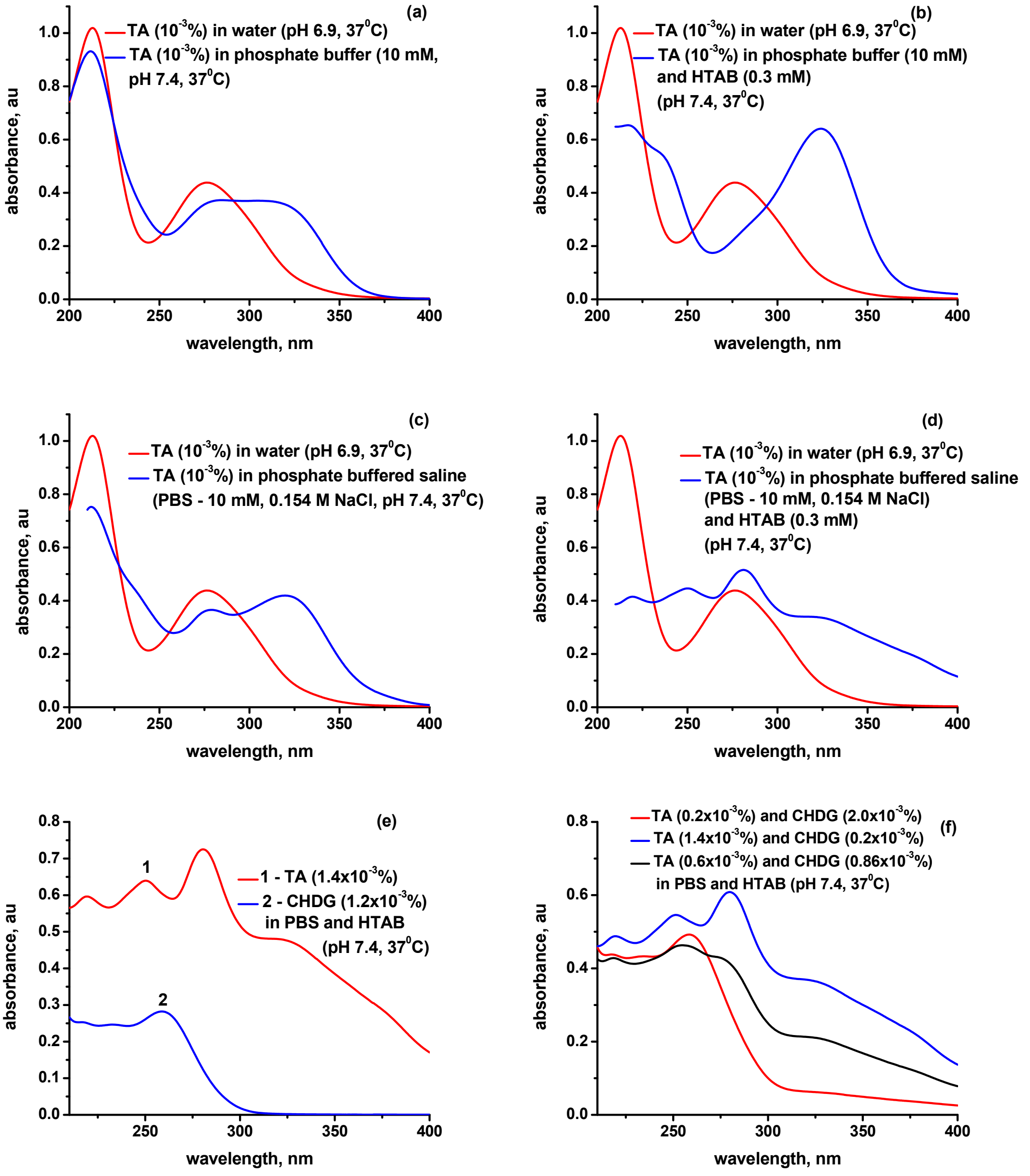

(18) Figure S8. Changes in UV-VIS spectra of TA in different aqueous environments (a-d), the corresponding spectra of TA and CHDG (taken separately) in PBS and HTAB (e) and the UV-VIS spectra for three TA-CHDG mixtures in the same release solution (PBS+HTAB) at the specified compositions (f).

(19) Experimental algorithm on determination of TA/CHDG concentrations in release medium

Prior to assess TA/CHDG concentrations in the release medium during the *in vitro* process, a matrix of concentration values for both components was built up with the aim to cover the majority of the compositions possible to be encountered in the operating experimental conditions (see Table S4). Any composition in the table expressed as the ratio of TA/CHDG concentrations should follow the Lambert-Beer's law by diluting correspondingly using the same solvent. Indeed, keeping the concentration ratio constant, all the systems obey Lambert-Beer's law (Figure S9).

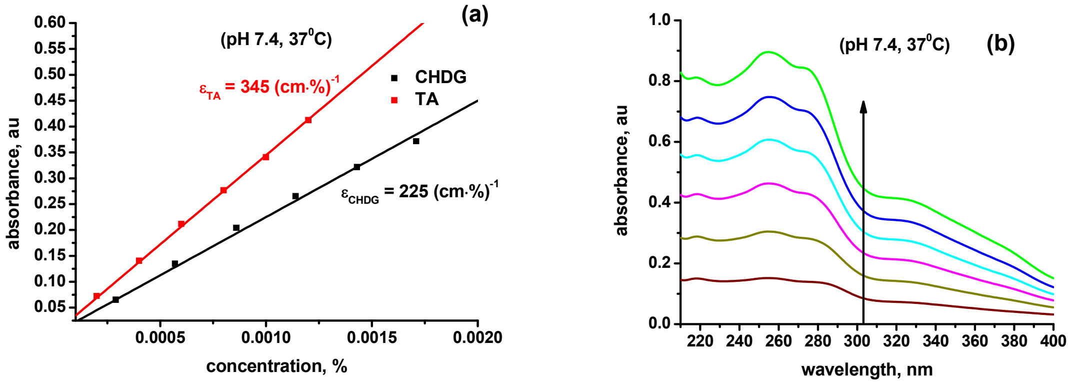

Figure S9. (a) Obeying Lambert-Beer's law starting from (b) an initial solution of minimum concentration of 2.03×10^{-4}% TA and 2.9×10^{-4}% CHDG and a final solution of maximum concentration of 1.20×10^{-3}% TA and 1.71×10^{-3}% CHDG in PBS and HTAB with keeping the concentration ratio constant (TA/CHDG – 0.7; the arrow indicates how components concentrations increase).

Once established these benchmarks, each experimental measurement has been processed as follows:

- from UV-VIS spectrum of a certain composition (extracted from the release medium at a desired time), the ratio of absorbances belonging to the two components (A_{325nm}/A_{260nm}), corrected by the corresponding extinction coefficients, was compared to the values from Table S4. The closest value found in the table defines the composition most likely to be in the real system (extracted from the release medium);
- based on the well-established ratio of AT/CHDG concentrations, a series of 5-6 solutions of known concentration, but having the same ratio, were used to generate a calibration curve (straight line) according to Lambert-Beer's law;
- finding out the TA and CHDG concentration in the real sample.

The same steps sequence has been followed for every sample extracted from the release solution in order to calculate the fractional release of TA and CHDG on time.

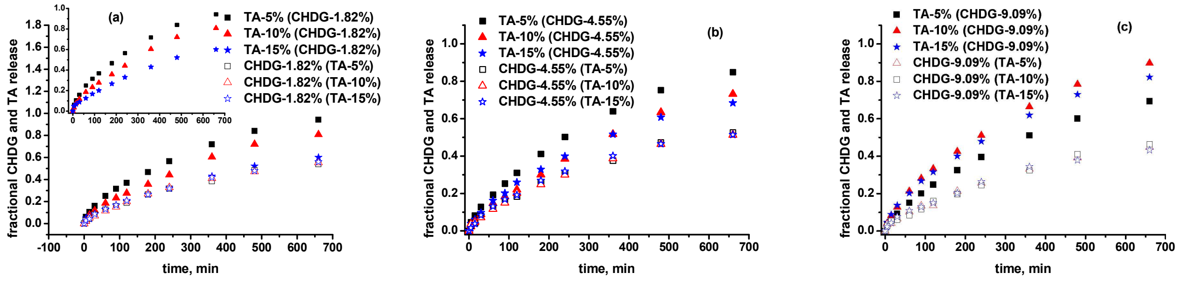

(20) Figure S10. TA (filled symbols) and CHDG (open symbols) release from collagen-based matrices into PBS-HTAB solution (pH 7.4) at 37^{0}C.

(21) Ionic strength and temperature effects on practical/apparent acid exponent pK_{ap}

Let HA^{Z+1} be a weakly acidic species giving rise to dissociation equilibrium as follows:

$$(HA)^{Z+1} \rightleftharpoons A^{Z} + H^{+} \tag{S2}$$

for which the thermodynamic (K_a) and apparent (K_{ap}) dissociation constants can be expressed as:

$$K_a = \frac{a_{H^+} \cdot a_{A^Z}}{a_{HA^{Z+1}}} \tag{S3}$$

$$K_{ap} = \frac{a_{H^+} \cdot [A^Z]}{[(HA)^{Z+1}]} \tag{S4}$$

where the square brackets denote the molarity of the species written inside them, and Z – an integer number - positive, negative or zero, denoting the net electrical charge on the species A. Knowing that the activity of a species i is given by the relationship:

$$a_i = \frac{c_i \cdot f_i}{c_o} \tag{S5}$$

where c_i and f_i are the molarity and activity coefficient of species i, respectively, and c_0 is strictly equal to 1 mol L^{-1} (taken for a standard state of system), it is easy to obtain the equation below:

$$pK_{ap} = pK_a + \log\left(\frac{f_{A^Z}}{f_{HA^{Z+1}}}\right) \tag{S6}$$

In equation (S6), $pK_a = -\log K_a$ and $pK_{ap} = -\log K_{ap}$.

On the other hand, the coefficient activity is strongly dependent on the ionic valency and the ionic strength. Thus, according to the Davies-type equations extensively used, the mean ionic activity coefficient of an electrolyte ($f_\pm$) in aqueous environment at 25^0C is given by [95,96]:

$$-\log f_\pm = 0.5 \cdot |Z_A \cdot Z_B| \cdot \left(\frac{\sqrt{\frac{I}{c_0}}}{1+\sqrt{\frac{I}{c_0}}} - a \cdot \sqrt{\frac{I}{c_0}}\right) \tag{S7}$$

where $Z_{A,B}$ denotes the valences of the constituent ions, c_0 - the same quantity as mentioned above, I – the ionic strength of solution, in mol·L^{-1} and a – a dimensionless constant of 0.3 [96]. Equation (S7) may be equally applied to both molar and molal concentrations [4] and is in a very good agreement with the experimental values of the measured ionic activity coefficients to a number of electrolytes at molalities up to 0.3 mol·kg^{-1} [97]. If I is considered dimensionless, but numerically equal to its value expressed in mol·L^{-1}, equation (S7) becomes:

$$-\log f_\pm = 0.5 \cdot |Z_A \cdot Z_B| \cdot \left(\frac{\sqrt{I}}{1+\sqrt{I}} - 0.3 \cdot \sqrt{I}\right) \tag{S8}$$

Starting from equation (S8) and supposing that $f_\pm = \left(f_A^x \cdot f_B^y\right)^{1/(x+y)}$ for a presumable compound $A_x^{Z_1} A_y^{Z_2}$, it is quite simple to obtain the following ionic strength dependence for the activity coefficient of every single species:

$$-\log f_i = 0.5 \cdot |Z_i^2| \cdot \left(\frac{\sqrt{I}}{1+\sqrt{I}} - 0.3 \cdot \sqrt{I}\right) \tag{S9}$$

with $i = A,B$. By combining equations (S6) and (S9), a useful relationship of the apparent acid exponent in term of the ionic strength (and ionic valency) may result:

$$pK_{ap} = pK_a + (2Z+1) \cdot 0.5 \cdot \left(\frac{\sqrt{I}}{1+\sqrt{I}} - 0.3 \cdot \sqrt{I}\right) \tag{S10}$$

where Z pertains to the dissociation equilibrium (S2). To assess the temperature influence on the thermodynamic dissociation constant K_a and, in turn, onto the apparent dissociation constant K_{ap}, it is advisable to take into account the relation written as:

$$dpK_a(t) = \left(\frac{dpK_a(t)}{dt}\right)\cdot dt \tag{S11}$$

or, after integration between the standard temperature of 25^{0}C and the working temperature t:

$$pK_a(t) = \int_{25^0C}^{t}\left(\frac{dpK_a(t)}{dt}\right)\cdot dt = pK_a(25^0\,C) + \left(\frac{dpK_a(t)}{dt}\right)\cdot(t-25^0\,C) \tag{S12}$$

$$pK_{ap}(t,I) = pK_a(25^0\,C) + \left(\frac{dpK_a(t)}{dt}\right)\cdot(t-25^0\,C) + (2Z+1)\cdot 0.5\cdot\left(\frac{\sqrt{I}}{1+\sqrt{I}} - 0.3\cdot\sqrt{I}\right) \tag{S13}$$

By logarithmizing, the equation (S4) can be rewritten as:

$$pH = pK_{ap} + \log\left(\frac{[A^Z]}{[(HA)^{Z+1}]}\right) \tag{S14}$$

The particular case of the phosphate buffer

$$H_2PO_4^- \rightleftharpoons HPO_4^{2-} + H^+ \tag{S15}$$

is associated to an adapted form of the equation (S14) at the operational pH of 7.40:

$$7.40 = pK_{ap} + \log\left(\frac{[HPO_4^{2-}]}{[H_2PO_4^-]}\right) \tag{S16}$$

where Z = -2. To determine accurately the values of both pK_{ap} and ionic strength based on the concentrations $[HPO_4^{2-}]$, $[H_2PO_4^-]$ and the additional amount of NaCl in solution (with NaCl considered fully dissociated and a final concentration of 0.154 M), a number of iterative steps was obeyed:

- estimating the first value of pK_{ap} according to equation (S13) by taking into account $pK_a(25^0C)$ = 7.20, $dpK_a/d\,t$ = -0.0028 1/^{0}C [58], I = 0.154 and t = 37^{0}C;
- inserting pK_{ap} value, as estimated above, in the equation (S16) to calculate $[HPO_4^{2-}]$, $[H_2PO_4^-]$ starting from the concentration of 10 mM of sodium phosphate buffer used;
- calculation of the corrected ionic strength by summing the contributions due to the buffer and NaCl in solution;
- returning iteratively to the first three steps three times (by considering each time the corrected value of the overall ionic strength calculated in the 3rd step) till the estimated values of pK_{ap} and the total ionic strength reach a good enough convergence.

According to this algorithm, I = 0.026 + 0.154 = 0.180 and pK_{ap}= 6.80.

(22) Relationships used for defining some quantities at equilibrium swelling required in the evaluation of crosslinking degree associated with the studied collagen-based matrices

$$Q^{eq}_{v,matrix} = [1/\rho_{matrix} + (Q^{eq}_{w,matrix} - 1)/\rho_{buffer}]\cdot\rho_{matrix} \tag{S17}$$

$$Q^{eq}_{w,coll} = Q^{eq}_{w,matrix}\cdot(1+x+y) - (x+y) \tag{S18}$$

$$Q^{eq}_{v,coll} = Q^{eq}_{v,matrix}\cdot\left(1 + \frac{x\cdot\rho_{coll}}{\rho_{TA}} + \frac{y\cdot\rho_{coll}}{\rho_{CHDG}}\right) - \left(\frac{x\cdot\rho_{coll}}{\rho_{TA}} + \frac{y\cdot\rho_{coll}}{\rho_{CHDG}}\right) \tag{S19}$$

$$\nu_{2eq} = 1/Q^{eq}_{v,coll} \tag{S20}$$

where $Q^{eq}_{w,matrix}$ is the equilibrium weight swelling ratio of the collagen-based matrix, $Q^{eq}_{w,coll}$ - the equilibrium weight swelling ratio corresponding to the collagen only, $Q^{eq}_{v,matrix}$ - the equilibrium volume swelling ratio of the collagen-based matrix, $Q^{eq}_{v,coll}$ - the equilibrium volume swelling ratio of the collagen only, ν_{2eq} - the collagen volume fraction in the swollen collagen-based matrix at

equilibrium, x – the weight fraction of TA with respect to collagen in the studied matrices, y – the weight fraction of CHDG with respect to collagen in the studied matrices, ρ_{matrix} – the density of collagen-based matrix with a certain composition, ρ_{coll} – the density of collagen matrix, ρ_{TA} (2.120 g/cm^3) – the density of TA and ρ_{CHDG} (1.135 g/cm^3) – the density of CHDG.

$$c_p = 17.6 \cdot \frac{\rho_{coll}}{Q^{eq}_{v,coll} \cdot M} = 17.6 \cdot \frac{v_{2eq} \cdot \rho_{coll}}{M} \qquad \text{(S21)}$$

$$\Delta c = \left(c_p^2 + 4c_e^2\right)^{1/2} - 2c_e \qquad \text{(S22)}$$

c_p – concentration of the negative net fixed charges on collagen (in mol/cm^3); c_e – half the overall concentration of the dissociated components of electrolyte found outside the matrix (0.180×10^{-3} mol/cm^3), namely 0.090×10^{-3} mol/cm^3.
